# Expression, Purification, and Characterization of a Well-Adapted Tyrosinase from Peatlands Identified by Partial Community Analysis

**DOI:** 10.1021/acs.est.1c02514

**Published:** 2021-06-22

**Authors:** Felix Panis, Rudolf F. Krachler, Regina Krachler, Annette Rompel

**Affiliations:** † Universität Wien, Fakultät für Chemie, Institut für Biophysikalische Chemie, Althanstraße 14, 1090 Wien, Austria; ‡ Fakultät für Chemie, Institut für Anorganische Chemie, Universität Wien, Althanstraße 14, 1090 Wien, Austria

**Keywords:** global warming, carbon cycle, enzyme, heterologous expression, kinetics

## Abstract

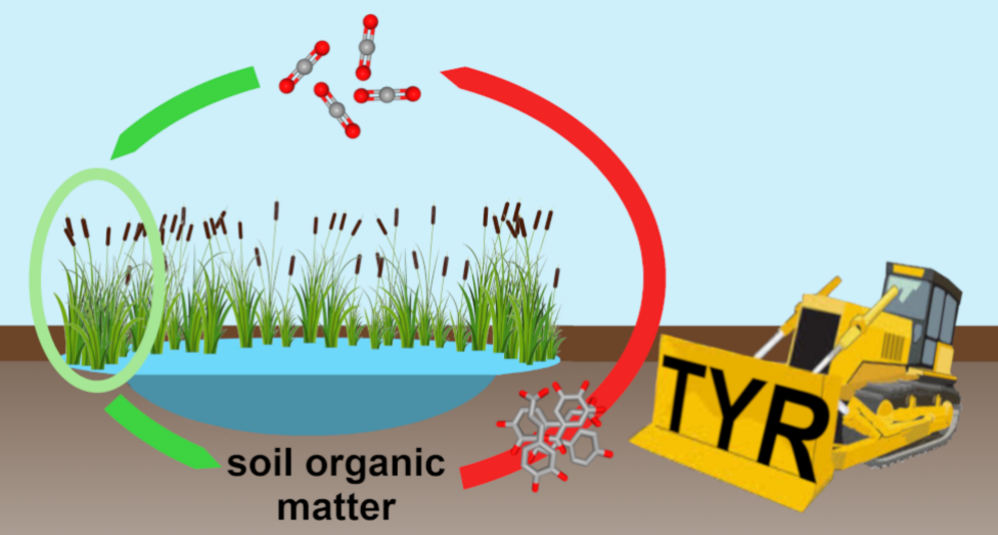

In peatlands, bacterial tyrosinases (TYRs) are proposed to act as key regulators of carbon storage by removing phenolic compounds, which inhibit the degradation of organic carbon. Historically, TYR activity has been blocked by anoxia resulting from persistent waterlogging; however, recent events of prolonged summer drought have boosted TYR activity and, consequently, the release of carbon stored in the form of organic compounds from peatlands. Since 30% of the global soil carbon stock is stored in peatlands, a profound understanding of the production and activity of TYRs is essential to assess the impact of carbon dioxide emitted from peatlands on climate change. TYR partial sequences identified by degenerated primers suggest a versatile TYR enzyme community naturally present in peatlands, which is produced by a phylogenetically diverse spectrum of bacteria, including Proteobacteria and Actinobacteria. One full-length sequence of an extracellular TYR (*Sz*TYR) identified from a soda-rich inland salt marsh has been heterologously expressed and purified. *Sz*TYR exhibits a molecular mass of 30 891.8 Da and shows a pH optimum of 9.0. Spectroscopic studies and kinetic investigations characterized *Sz*TYR as a tyrosinase and proved its activity toward monophenols (coumaric acid), diphenols (caffeic acid, protocatechuic acid), and triphenols (gallic acid) naturally present in peatlands.

## Abstract 2

The diversity of the tyrosinase community present in peatlands has been investigated and the first tyrosinase indigenous to peatlands has been expressed, purified, and biochemically characterized.

## Introduction

Northern peatlands cover 3% of the land area^1^ but constitute 30% of the global soil carbon stock,^2,3^ equivalent to 60% of the global atmospheric carbon pool.^4^ They represent an unbalanced system and have accumulated vast amounts of carbon since the last glacial maximum by sequestering CO_2_ from the atmosphere and storing it as organic carbon at a higher rate than releasing it.^2,5^ The main part of organic carbon in peatlands is stored in the form of humic substances, a structurally heterogeneous group of polyaromatic, recalcitrant polymers.^6^ The oxidative breakdown of humic substances leads either to carbon release (in the form of CO_2_) into the atmosphere or to the production of soluble, small phenolic compounds, which are slowly discharged *via* rivers and aquifers.^7,8^ Besides the degradation of humic substances, small phenolic compounds can be released by peatland-specific vegetation, either actively or as a result of cell lysis.^9^ The resulting abundance of phenolic compounds in peat is inhibitory to the growth of microorganisms and the activity of extracellular enzymes, which are responsible for the degradation of organic matter (*e.g*., β-glucosidases, peroxidases, xylosidases, and chitinases),^10−13^ with only a few enzymes, such as tyrosinases (TYRs), capable of removing phenolic compounds *via* hydroxylation, oxidation, and subsequent spontaneous polymerization.

**Figure 1:**
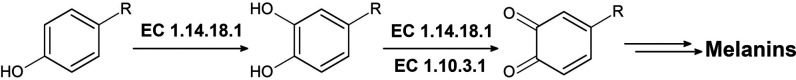
Reactions catalyzed by TYRs. Monophenols are hydroxylated in the ortho-position (monophenolase activity), followed by the oxidation of *o*-diphenols to *o*-quinones (diphenolase activity), which undergo nonenzymatic polymerization to form high-molecular-weight melanins. The figure has been edited using GIMP 2.10.18 (https://www.gimp.org).

Tyrosinases (TYRs) belong to the type III copper enzyme family and are ubiquitously distributed in nature among archaea,^14^ bacteria,^15^ fungi,^16^ plants,^17−20^ and animals, including mammals.^21^ Using molecular oxygen, TYRs catalyze the ortho-hydroxylation of monophenols to *o*-diphenols (monophenolase activity, EC 1.14.18.1) and the subsequent oxidation of *o*-diphenols to *o*-quinones (diphenolase activity, EC 1.10.3.1), which rapidly undergo nonenzymatic reactions, resulting in the formation of brown-black melanins, a group of high-molecular-weight polymers (Figure 1).^15,16,22,23^

TYRs feature a di-copper center with each copper ion coordinated by three conserved histidine residues^24^ (Figure S1). In solution, the active centers of TYRs are predominantly (85%) present in the oxygen-free state (the met-form).^25^ Upon binding of dioxygen, the active center is converted into the oxy-form, which exhibits a characteristic charge transfer band at 345 nm.^26,27^*In vivo*, oxidation of diphenols converts the met-form into the oxy-form, while *in vitro*, H_2_O_2_ can be used alternatively to form the oxy-complex.^28^ In TYRs, a variable frame of second shell amino acids is responsible for the enzyme’s substrate preferences.^24,29,30^ Especially, two nonconserved amino acid positions located next to the first and second CuB-coordinating histidine (Figure S1) and termed first (His_B1_ + 1) and second (His_B2_ + 1) activity controller were shown to govern substrate binding and orientation in TYR enzymes.^18,20,31−37^*In vivo*, most TYRs are expressed as latent proenzymes.^24^ In TYRs from plants,^22^ fungi,^16^ and some bacteria,^15^ the latency is caused by the C-terminal domain of the TYR enzyme,^24^ while in TYRs from the bacterial genus *Streptomyces* a separate, so-called caddie protein^15,38^ is present, both of which sterically block access to the active center.

TYRs are capable of removing phenolic compounds by initiating their polymerization (see Figure 1) and, therefore, proposedly play a central role in the storage and release of organic carbon in peatlands.^13^ Historically, the activity of TYRs in peatlands has been suppressed by oxygen deprivation due to high water levels. Accordingly, anoxia resulting from persistent waterlogging has been proposed to act as a key regulator of peatland stability.^10^ However, in the wake of climate change, an increase in the frequency and duration of summer droughts has become a likely scenario, which will promote the aeration of previously anoxic peat layers and will consequently boost TYR activity. It is proposed that the concentration of small phenolic compounds will consequently decrease and their inhibitory effect on organic matter degrading enzymes will recede, which, in turn, will lead to the release of vast amounts of stored organic carbon (in the form of CO_2_) into the atmosphere,^10,39^ which will further promote climate change.

The microbial community in peatland soils is dominated by bacteria, both in numbers and as far as the influence on carbon cycling is concerned (while archaea and fungi are less prevalent),^40−45^ and, consequently, tyrosinase activity in peatlands is likely to be equally dominated by bacterial TYR enzymes as well. The species-rich bacterial genus of *Streptomyces* has been particularly associated with plant necromass degradation.^46^ In many *Streptomyces* species, a MelC operon is present, which harbors the MelC2 gene coding for an ∼30 kDa tyrosinase enzyme.^15^ A second gene coding for the so-called caddie protein (MelC1) is located upstream of the MelC2 gene (Figure S2). *In vivo*, the two gene products are polycistronically expressed and their products form a heterodimeric complex.^47,48^ The TYR enzyme (MelC2) carries the active center and is responsible for the enzymatic activity, while the caddie protein (MelC1) is required for correct folding of the TYR enzyme and copper incorporation into the active center.^49−51^ Moreover, a twin-arginine motif (a stretch of consecutive amino acids with the consensus sequence S/T-R-R-X-F-L-K^52^) is located close to the N-terminus of the caddie protein, thus allowing efficient secretion of the complex (TYR–caddie protein) *via* the twin-arginine translocation (TAT) secretion pathway.^15,53^

Despite the potentially decisive influence of TYRs produced by indigenous soil bacteria on the fine balance of carbon storage and release in peatlands,^10^ no research targeting the subcommunity of TRY-producing bacteria has been conducted so far. Moreover, no information about the catalytic properties of TYR enzymes present in peatlands has been reported to date. In this study, the abundance and diversity of bacterial TYR enzymes in peat have been investigated using metagenomic DNA extracted from peat samples as a template. Degenerated TYR primers, which bind to conserved DNA regions coding for the CuA- and CuB-coordinating sites of bacterial TYRs (Figures S1 and S3), selectively amplified bacterial TYR partial sequences. We present, to the best of our knowledge, the first partial community analysis of bacterial TYRs present in peat samples. Sequencing of TYR partial sequences (covering the regions between the CuA- and CuB-coordinating sites) in combination with a BLAST search^54^ revealed a diverse spectrum of bacterial TYR genes, potentially originating from different bacterial phyla (Proteobacteria and Actinobacteria), with highly heterogeneous amino acid sequences. The full-length sequence of one bacterial TYR (from *Streptomyces* sp. ZL-24) has been identified from metagenomic DNA extracted from peat samples to investigate its biochemical characteristics. After heterologous expression and purification, the corresponding enzyme (*Sz*TYR) reveals a TYR that is designed for secretion, highly adapted to the ambient environmental conditions, and active on a broad spectrum of phenolic substrates (tyramine, l-tyrosine, dopamine, l-3,4-dihydroxyphenylalanine (l-DOPA), coumaric acid, caffeic acid, protocatechuic acid, gallic acid). In the face of climate change, we hope that the results presented herein will stimulate research into the environmental impact of TYRs present in various ecosystems.

## Results and Discussion

### Metagenomic DNA Extraction and Purification

Metagenomic DNA was extracted to identify TYR genes from soil organisms. Since humic substances are formed by the condensation of low-molecular-weight phenolic compounds by phenol oxidases, such as TYRs,^6^ samples were taken from peat soils rich in humic substances (see Materials and Methods), as indicated by their characteristic black-brown color.

A DNA extraction protocol adapted to efficiently remove humic substances was developed (see Materials and Methods) and yielded maximum amounts of metagenomic DNA ranging from 23 to 27 μg/g of peat. The quality of the metagenomic DNA extract was assessed by agarose gel electrophoresis, which showed a sharp band at ∼20 kbp (Figure S4).

### Degenerated Primer Design

**Figure 2:**
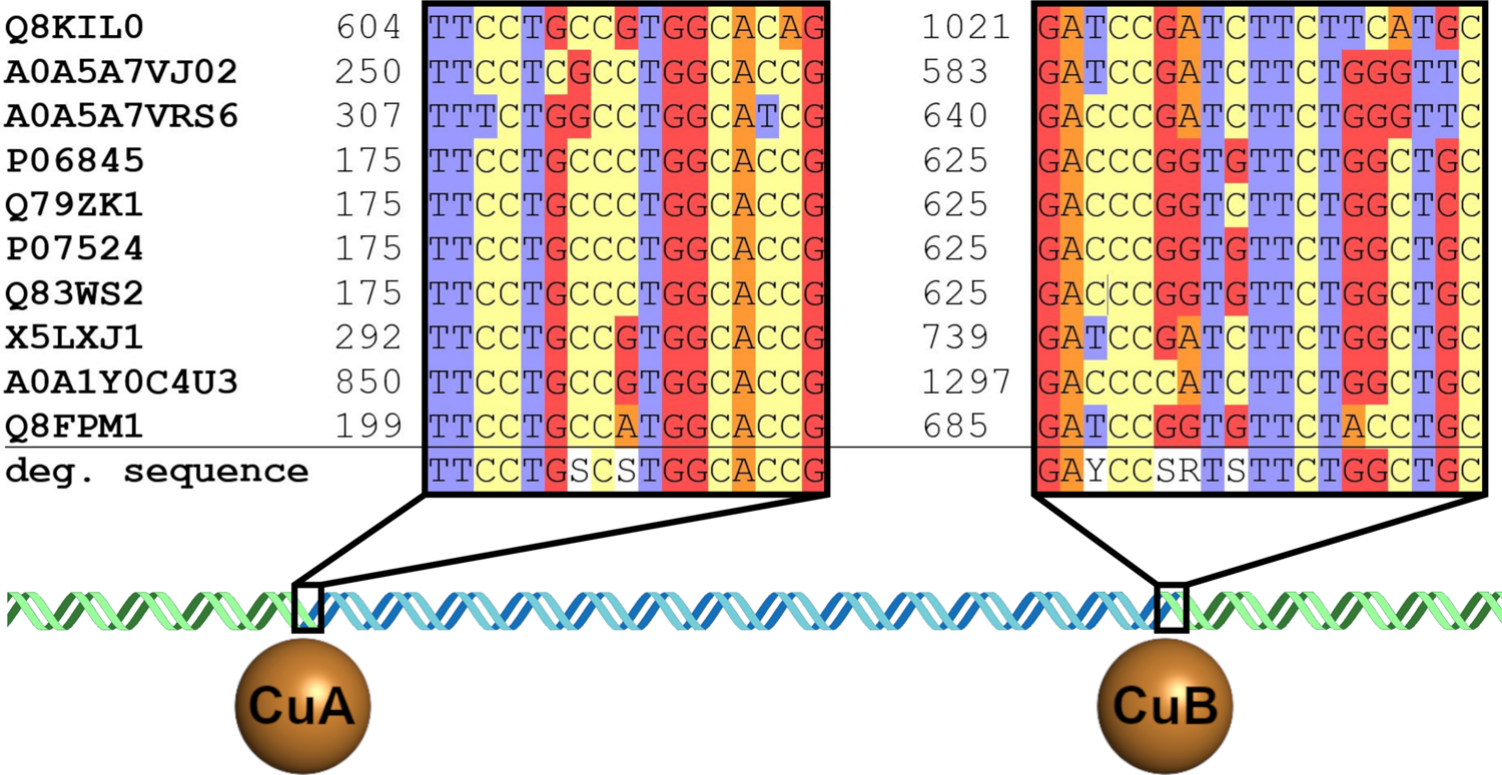
Binding regions of the degenerated type III copper protein primers. Aligned template TYR nucleotide sequences with UniProt^60^ identifiers (listed on the left in bold letters) and the location of the sequences within the respective gene (indicated by the numbers on the left side of the sequences). “deg. sequence” indicates the sequence used for the degenerated forward primer and the degenerated reverse primer. For the reverse primer, the reverse complement of the displayed sequence was used (Table S1). The double helix represents a bacterial full-length TYR gene. The blue part between the primer binding regions represents the partial sequence amplified by the degenerated type III copper protein primers, and the green parts represent the obscure parts of the TYR gene outside of the amplified partial sequence. The brown spheres labeled CuA and CuB represent the location of the sequences coding for the CuA- and CuB-coordinating amino acids. The figure has been edited using GIMP 2.10.18 (https://www.gimp.org).

The regions coding for the C-terminus and the N-terminus of TYRs show little conservation and are thus unapt for the amplification of unknown TYR sequences. In contrast, the CuA- and CuB-coordinating regions (Figures S1 and S3) show a high level of conservation combined with a high level of selectivity for type III copper proteins. Since bacteria are most strongly associated with carbon cycling among the microbial community in peatlands, primers targeting bacterial TYRs were designed. Proteobacteria and Actinobacteria are TYR-producing phyla particularly abundant in peatland soils.^40,41,46^ Thus, multiple proteobacterial (including the genera *Rhizobium*(55,56) and *Rhodanobacter*(57)) and actinobacterial (including the genera *Streptomyces*,^58^*Mycobacterium*,^59,60^ and *Corynebacterium*(46)) full-length TYR sequences of bacteria indigenous to soil were included in the alignment (Figure 2) and searched for regions showing a high level of conservation over a distance suitable for primer binding (approximately 20 base pairs in length, which allows specific annealing during polymerase chain reaction (PCR) experiments). A set of primers (fwd: 4-fold degenerated; rev: 16-fold degenerated, Table S1) was designed binding to two conserved sites (fwd: 17 base pairs; rev: 19 base pairs) in the nucleotide sequences of proteobacterial and actinobacterial TYRs, which are located within the regions coding for the CuA- and CuB-coordinating amino acids of the template sequences (Figures 2, S2, and S3).

### Genetic and Structural Heterogeneity of Partial Tyrosinase Sequences Reveals a Diverse Tyrosinase Community

PCR experiments using degenerated type III copper protein primers binding to metagenomic DNA extracted from peat produced 350–550 base pair amplicons, which are in accordance with the expected length of the nucleotide sequence between the CuA- and CuB-coordinating regions.^61−63^ Nineteen TYR partial sequences (Table S2) were identified by sequencing which covered the region between the primer binding sites (Figures 2, S2, S3, and Table S1).

A BLAST search (see the Supporting Information) revealed that sequence 19 was identical (100% identity; Tables S2 and S3) to the sequence of a putative TYR from *Streptomyces* sp. ZL-24 (A0A2S3Y8X7), which has been identified by a genome sequencing project focusing on the identification of *Streptomyces* species in a wetland.^64^ However, except for the nucleotide sequence, no further information about the putative TYR A0A2S3Y8X7 is available to date. Moreover, six partial sequences showed sequence identity levels of more than 75% to previously described proteobacterial and actinobacterial TYRs (Table S3), while for 12 partial sequences only low-identity matches (40.0–65.8%, sequences 1–8 and 15–18) were identified. A phylogenetic alignment of the identified partial sequences (Figure S5) in combination with the results of the BLAST search (Table S3) suggests that the identified sequences originate from various species and belong to different bacterial phyla (Proteobacteria and Actinobacteria).

The low level of genetic conservation leads to a correspondingly high level of diversity in the amino acid sequences (Table S4). Pairwise amino acid sequence alignment of the 19 identified putative TYR partial sequences revealed identity values ranging from 12.2% (sequences 6 and 10) to 83.2% (sequences 2 and 5, Table S5). Situated within the region amplified by the degenerated type III copper protein primers are the activity controllers (first activity controller: His_B1_ + 1, second activity controller: His_B2_ + 1; Figures S1 and S3), which display a high level of heterogeneity in the 19 sequences identified herein. Four amino acids (Asn, Asp, Ser, Gly) are featured in the position of the first activity controller (His_B1_ + 1) and seven amino acids (Asn, Ile, Gly, Arg, Val, Thr, Met) are featured in the position of the second activity controller (His_B2_ + 1), realized as eight different combinations of both activity controllers (Figure S6).

Enzymatic activity, substrate preferences, and pH dependency of TYRs are controlled by a framework of second shell amino acids located in and around the catalytic pocket.^24,33,50,65,66^ In bacterial TYRs, mono- and diphenolase activities have been observed for all investigated enzymes so far; however, the first (His_B1_ + 1) and second (His_B2_ + 1) activity controllers critically influence the kinetic behavior and substrate acceptance.^18,20,34,37,67^ The high level of heterogeneity in both the overall TYR sequences and the amino acids featured in the position of the activity controller residues suggests that TYR enzymes present in peatlands act on a broad scope of substrates and, therefore, have the potential of efficiently removing phenolic compounds with increased aeration of previously anoxic peat layers. In addition, the degenerated type III copper protein primers designed within this study represent a valuable tool for further partial community analysis of TYR-producing organism *via*, *e.g*., single-strand conformation polymorphism (SSCP) and denaturing gradient gel electrophoresis (DGGE).

### Identification of a Full-Length Tyrosinase Sequence

To gain a deeper understanding of the biochemical properties of TYRs present in peatland soil, we focused on the identification of the full-length sequence of partial sequence 19, which showed 100% identity to the sequence of the putative tyrosinase from *Streptomyces* sp. ZL-24 (A0A2S3Y8X7, sequence 19). Therefore, a reverse primer (Table S1) binding to the C-terminus of the A0A2S3Y8X7 protein (MelC2, TYR) deposited in the UniProtKB database was designed. The forward primer (Table S1) was designed to bind to the N-terminus of the A0A2S3Y8X5 protein (MelC1, caddie protein) deposited in the UniProtKB database, which is located upstream of the gene for A0A2S3Y8X7 (MelC2, TYR) in the genome of *Streptomyces* sp. ZL-24 (GenBank accession MTHF01000004).^64^ Metagenomic DNA extracted from peat samples served as the template. Sequencing revealed that the pair of primers produced a MelC operon as it is commonly found in *Streptomyces* species: a 396 base pair open reading frame (ORF) coding for a caddie protein (MelC1) is followed by an 828 base pair ORF coding for a tyrosinase (MelC2), with a short noncoding sequence (59 base pairs) interspaced between MelC1 and MelC2 (Figure S2 and Table S6). The nucleotide sequence of the caddie protein (MelC1) identified herein showed an identity level of 100% to the nucleotide sequence of A0A2S3Y8X5 (Table S3), while the nucleotide sequence of the tyrosinase gene (MelC2) identified herein exhibited one silent mutation (T135C) compared to A0A2S3Y8X7.^64^ For simplicity, the protein encoded by the full-length sequence of the partial TYR sequence 19 identified herein (originating from *Streptomyces* sp. ZL-24) will in the following discussion be addressed as “*Sz*TYR”.

Additionally, a 16S RNA analysis semiselectively targeting sequences from *Streptomyces* species (see Materials and Methods, Supporting Information) identified a 16S RNA sequence exhibiting 98.92% identity to the 16S RNA sequence from *Streptomyces* sp. ZL-24 (Table S7). Metagenomic DNA extracted from peat samples served as a template. Since 16S RNA sequences showing more than 98.65% identity are assumed to stem from the same species,^68^ it can be concluded that *Streptomyces* sp. ZL-24 is present in the peat samples investigated herein.

### Recombinant Expression and Purification of *Sz*TYR

Heterologous expression of the *Sz*TYR gene in *Escherichia coli* BL21(DE3) was attempted. Various reports demonstrated that the expression of active *Streptomyces* tyrosinase requires the coexpression of soluble MelC1 (caddie protein).^61,63,69^ Consequently, different expression systems for the concomitant expression of MelC1 (caddie protein) and MelC2 (tyrosinase) were evaluated. Following previous reports,^69^ the expression of MelC1 and MelC2 under the control of separate *tac*-promotors led to an increase in the yield of active tyrosinase compared to the expression of the polycistronic MelC operon (containing MelC1 and MelC2) under the control of a single *tac*-promotor, as indicated by increased discoloration of the expression medium. Moreover, codon optimization toward the codon usage of *E. coli* of MelC1 (caddie protein) further increased the production of active *Streptomyces* tyrosinase^63^ and led to measurable tyrosinase activity levels of the expression medium using the substrate dopamine (Figure S7C). Moreover, enzymatic activity was increased 170-fold by fusing the codon-optimized caddie protein to a glutathione *S*-transferase (GST)-tag (compared to the codon-optimized caddie protein without a GST-tag). Enzymatic activity was predominantly (98%) located in the extracellular fraction, while the intracellular fraction displayed only marginal enzymatic activity. The catalytic activity of the extracellular fraction was 2 orders of magnitude higher when 0.5 mM CuSO_4_ was added to the expression medium (compared to protein expression without additional Cu^2+^). Copper incorporation into the active center of *Sz*TYR was assessed photometrically (see Materials and Methods) and revealed 1.4 Cu ions per active site. In comparison, the TYR isolated from *Streptomyces glaucescens* contained 1.8 Cu ions per active site,^31^ while for recombinantly expressed TYRs, 0.8 (*jr*PPO1)^37^ to 2.0 (*Bm*TYR)^66,70^ copper ions per active site have been reported previously.

The secretion of the heterodimeric complex (*Sz*TYR–caddie protein) is effectuated by a twin-arginine motive (Thr5-Arg6-Arg7-His8-Ala9-Leu10-Gly11) of the caddie protein. Since the caddie protein dissociates from the tyrosinase and aggregates once it has completed its assisting role during the folding process, the secretion process, and the incorporation of copper ions into the active center,^49−51^ the GST-tag attached to the caddie protein does not aid in the purification of the target protein. The increased expression yield achieved by fusing the caddie protein to a GST-tag can be attributed to the solubility-enhancing effects of the GST-tag, its chaperone-like function, and reduced enzymatic degradation.^71,72^ Thus, the GST-tag plays a crucial role in the production of soluble caddie protein, which is a prerequisite for the production of soluble and active *Sz*TYR. We suggest that this strategy can be employed to increase the expression yields of TYRs from various *Streptomyces* species. Expression at low temperatures (19 °C) proved adequate for the production of the soluble enzyme, which is in accordance with previous reports from TYRs from *Juglans regia* (20 °C),^20^*Malus domestica* (20 °C),^18^ and *Streptomyces avermitilis* (18 °C).^63^

**Figure 3:**
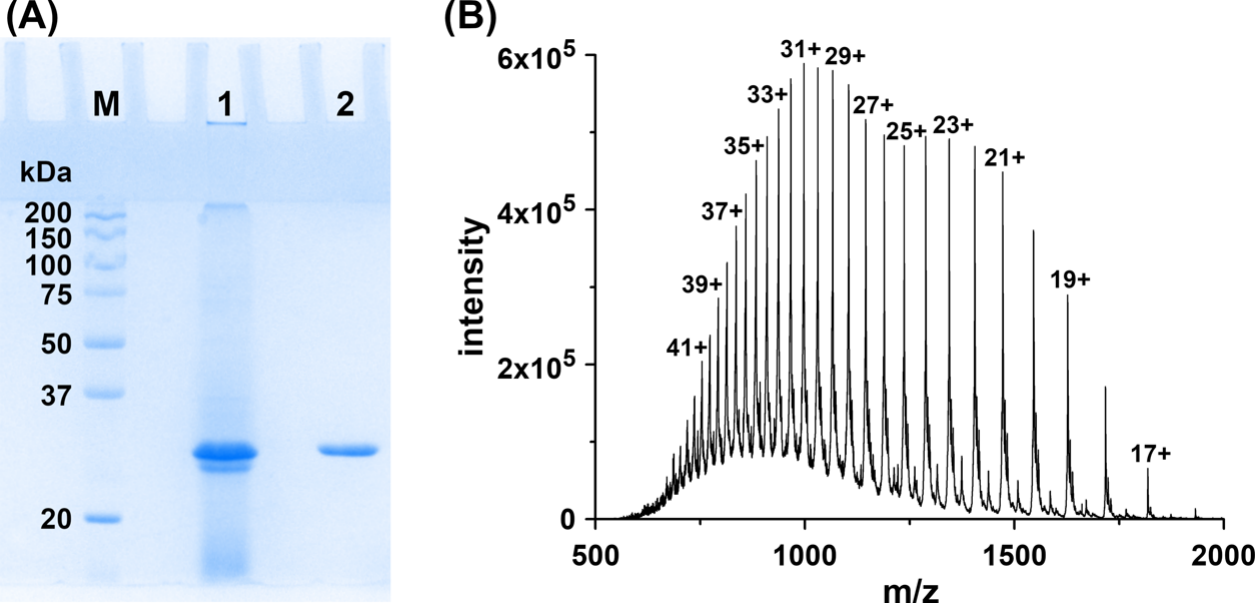
SDS-PAGE (12.5%) (A) and positive mode ESI-LTQ-Orbitrap Velos mass spectrum (B) of *Sz*TYR. (A) 1 represents the sample after ammonium sulfate precipitation and 2 represents the sample after additional purification *via* a MonoQ anion exchange column (Figure S8). The band at ∼31 kDa represents *Sz*TYR. Precision Plus Protein Standard Dual Color (Bio-Rad) was used as a marker (M). The gel has been cropped to the lanes of interest. (B) The calculated and measured masses of the protein are listed in Table S8. The peak labels correspond to the charge state (*z*) of the respective peaks. Detailed information about the experimental setup is provided in the Materials and Methods section. The figure has been edited using GIMP 2.10.18 (https://www.gimp.org).

A time-efficient (5 h) and highly effective purification protocol (purity level: 99% as indicated by sodium dodecyl sulfate-polyacrylamide gel electrophoresis (SDS-PAGE) and electrospray ionization mass spectrometry (ESI-MS), Figure 3) was developed for *Sz*TYR. Ammonium sulfate precipitation of the expression medium in combination with anion-exchange chromatography (MonoQ) efficiently removed protein contaminations as well as melanins produced during the expression process, which both bound to the column, while the tyrosinase enzyme passed through the column and was collected with the flow-through (Figure S8). TYR enzymes from several *Streptomyces* species have been expressed and purified previously.^63,69,73−75^ The highest expression yields were reported for TYR from *Streptomyces castaneoglobisporus* heterologously expressed in *E. coli* BL21(DE3)-pLysS (12 mg/L expression culture)^69^ and homologously overexpressed TYR from *Streptomyces antibioticus* (variable amounts of 10–20 mg/L of expression culture).^74^ Notably, the expression protocol reported herein produced 24 mg of active and purified tyrosinase per liter of expression culture.

### Molecular Mass Determination Reveals N-Terminal Methionine Processing for *Sz*TYR

ESI-LTQ-MS revealed a molecular mass of 30 891.8 Da, which matched with the calculated mass for the recombinantly expressed *Sz*TYR without the initial methionine residue (30 892.3 Da; Table S8 and Figure 3). N-Terminal methionine processing is a process commonly observed in *E. coli* and is caused by the enzyme methionine aminopeptidase (MetAP).^76^ Enzymatic cleavage by MetAP is determined by the size of the side chains adjacent to the first methionine and follows a simple rule: the initial methionine is cleaved if the side chains of the following residues have a radius of gyration of 1.29 Å or less.^77^*Sz*TYR investigated herein displays threonine (Thr2) and valine (Val3) adjacent to the initial methionine (Met1), with radii of gyration of 1.24 and 1.29, respectively. Thus, processing of the initial methionine is expected.

**Figure 4:**
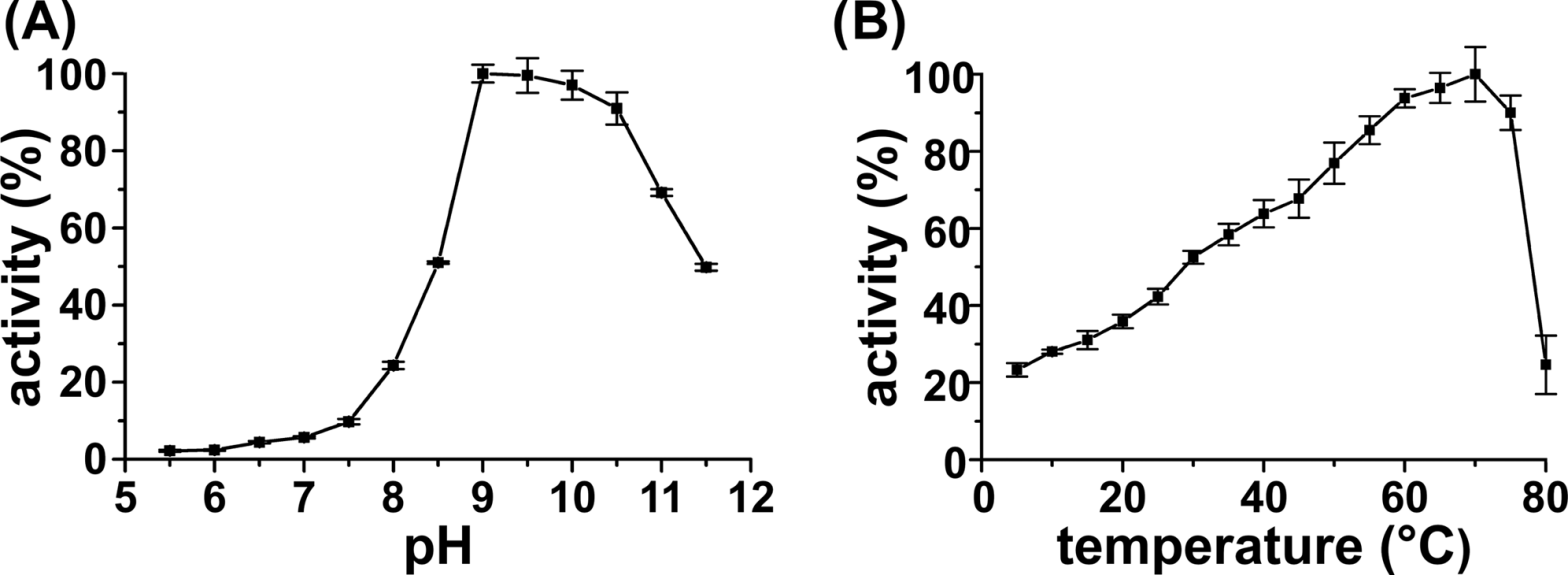
pH profile (A) and temperature profile (B) of *Sz*TYR using tyramine as a substrate. Measurements were performed in triplicate. The error bars indicate 1 standard deviation. Detailed information about the experimental setup is provided in the Materials and Methods section. The figure has been edited using GIMP 2.10.18 (https://www.gimp.org).

### Biochemical Characterization of *Sz*TYR

Spectroscopic investigations of *Sz*TYR demonstrated that the addition of H_2_O_2_ led to the formation of an absorption band at 345 nm, which corresponds to an O_2_^2–^ → Cu(II) charge transfer band characteristic for oxygen-bound type III copper centers. Saturation was reached after the addition of 2 molar equiv of H_2_O_2_ (Figure S9). Similar results have been reported for type III copper proteins from walnut (*jr*PPO1: 2 equiv^78^), sweet potato (*ib*CO: 2 equiv^79^), and *Melissa officinalis* (*mo*CO: 2 equiv^80^). Thus, the formation of the oxy-form proves the presence of a type III copper center in *Sz*TYR.

The pH profile of *Sz*TYR was investigated using tyramine (Figure S7A) as a substrate and revealed an unusually high pH optimum of pH 9.0 while retaining 50% activity at pH 11.5 (Figure 4). A monophenolic substrate (tyramine) was chosen for the determination of the pH optimum since diphenolic substrates show high levels of auto-oxidation at basic pH values.^81^ Most bacterial TYRs display pH optima between pH 6 and 7.^25^*Bt*TYR (*Bacillus thuringiensis*) displays a pH optimum of 9.0; however, no activity was detectable at pH 9.5 or higher.^82^ Interestingly, the sequence of *Sz*TYR was collected from a peat layer in the reed belt of a shallow soda lake, which displays a pH value of 9–9.5.^83,84^ Thus, we assume that organisms indigenous to this environment adapted over the last millennia to the prevalent, high pH values, which is reflected by the pH profile of *Sz*TYR. Further kinetic measurements were thus performed at the pH optimum of 9.0.

The temperature profile of *Sz*TYR was investigated using tyramine as a substrate and revealed increasing activity levels up to 70 °C with a sharp decrease in activity above 75 °C. The thermal stability of the enzyme was determined by a thermofluor assay, which revealed a *T*_m_ value of 67.4 °C (at pH 9.0) for *Sz*TYR (see Table S9 and Figure S10, Supporting Information). A temperature optimum of 65 and 70 °C has already been reported for the extracellular TYR from *Thermothelomyces thermophila*(83) and TYR from *B. thuringiensis*,^82^ respectively. We speculate that the pH value of 9.0–9.5 and high salinities (21–75 g/kg)^84^ measured at the sample point in combination with low O_2_ concentrations^2,85^ and the high concentrations of phenolic and organic acids characteristic for peatlands^11^ cause increased denaturing stress and create a hostile environment for extracellular enzymes. Consequently, robust enzymes with a sufficiently long lifetime are required to generate an evolutionary benefit for their host organism, which is potentially reflected by the pH and the temperature optimum of *Sz*TYR.

The catalytic behavior of *Sz*TYR was investigated using the standard substrates tyramine, l-tyrosine, dopamine, and l-DOPA. *Sz*TYR was confirmed as a TYR as it accepted both monophonic substrates (tyramine and l-tyrosine). However, activity (*k*_cat_ values) toward the diphenolic substrates (*k*_cat_ dopamine: 320 s^–1^, *k*_cat_l-DOPA: 520 s^–1^) was substantially higher than that toward monophenolic substrates (*k*_cat_ tyramine: 6.3 s^–1^, *k*_cat_l-tyrosine: 4.8 s^–1^). Accordingly, efficiency values (*k*_cat_/*K*_m_) were higher for diphenolic substrates (*k*_cat_/*K*_m_ dopamine: 43 s^–1^ mM^–1^, *k*_cat_/*K*_m_l-DOPA: 34 s^–1^ mM^–1^) than those for monophenolic substrates (*k*_cat_/*K*_m_ tyramine: 1.1 s^–1^ mM^–1^, *k*_cat_/*K*_m_l-tyrosine: 7.9 s^–1^ mM^–1^). Therefore, it can be assumed that, *in vivo*, *Sz*TYR targets diphenolic substrates (Table 1). The same trend has already been reported for TYR from *S. castaneoglobisporus* (*k*_cat_l-tyrosine: 4 s^–1^, *k*_cat_l-DOPA: 44 s^–1^)^70^ and TYR from *S. avermitilis* (*k*_cat_l-tyrosine: 0.58 s^–1^, *k*_cat_l-DOPA: 5.4 s^–1^),^63^ which both preferred diphenols over monophenols.

**Table 1:** Kinetic Parameters of *Sz*TYR with Standard Substrates ± 1 Standard Deviation^a^

substrate	*k*_cat_ (s^–1^)	*K*_m_ (mM)	*k*_cat_/*K*_m_ (s^–1^ mM^–1^)
monophenolic substrates			
tyramine	6.3 ± 0.27	5.6 ± 0.90	1.1 ± 0.19
l-tyrosine	4.8 ± 0.23	0.60 ± 0.074	7.9 ± 1.0
*p-*coumaric acid	2.6 ± 0.17	0.19 ± 0.037	13 ± 2.7
diphenolic substrates			
dopamine	320 ± 13	7.4 ± 0.95	43 ± 5.8
l-DOPA	520 ± 80	15 ± 3.7	34 ± 9.8
caffeic acid	630 ± 37	1.1 ± 0.22	570 ± 120
protocatechuic acid	59 ± 5.3	45 ± 7.9	1.3 ± 0.26
triphenolic substrates			
gallic acid	7.7 ± 0.83	6.9 ± 1.2	1.1 ± 0.23

a Measurements were performed in triplicate. Detailed information about the experimental setup is provided in the Materials and Methods section.

To further investigate the potential impact of *Sz*TYR on its natural environment, the activity of *Sz*TYR toward phenolic compounds present in peatlands^86,87^ was assessed. Monophenolic (*p*-coumaric acid, Figure S7E), diphenolic (caffeic acid, protocatechuic acid; Figure S7F,G), triphenolic (gallic acid, Figure S7H), and methoxylated phenolic substrates (ferulic acid, vanillic acid, vanillin; Figure S7I–K) were included in a substrate scope assay which revealed enzymatic activity toward *p*-coumaric acid, protocatechuic acid, caffeic acid, and gallic acid, whereas phenolic substrates carrying an *o*-methoxy group were not accepted as a substrate (Figure S11). Kinetic parameters (*k*_cat_ and *K*_m_ values) were determined for active substrates (*p*-coumaric acid, caffeic acid, protocatechuic acid, and gallic acid) and again revealed higher *k*_cat_ values for diphenolic substrates (*k*_cat_ caffeic acid: 630 s^–1^, *k*_cat_ protocatechuic acid: 59 s^–1^) than for monophenolic (*k*_cat_*p*-coumaric acid: 2.6 s^–1^) or triphenolic ones (*k*_cat_ gallic acid: 7.7 s^–1^). Consequently, *Sz*TYR produced by an indigenous soil bacterium (*Streptomyces* sp.) and physiologically secreted into its environment displays the potential of efficiently removing monophenolic, diphenolic, and triphenolic compounds naturally present in peatlands by copolymerization (see Figure S12, Supporting Information), thus reducing their inhibitory effect on soil organic matter degrading enzymes.

## Materials and Methods

### Metagenomic DNA Extraction and Purification

Two peat samples were collected at the following locations: 48°30′18.0″N 14°51′43.3″E (Tanner Moor, an acidic raised bog in Upper Austria, Austria, September 2017) and 47°45′13.2″N 16°44′55.5″E (a soda-rich inland salt marsh in the riparian zone of Lake Neusiedl, Burgenland, Austria, July 2020) and used for metagenomic DNA extraction (for detailed information on the sampling site see the Supporting Information).

One gram of frozen soil sample was thawed by incubation at room temperature and washed five times by suspension in 5 mL of a humic substance removal solution (200 mM Tris–HCl pH 9.0, 100 mM ethylenediaminetetraacetic acid (EDTA), 100 mM NaCl, 1% (m/v) poly(vinylpyrrolidone), 0.05% (v/v) Triton X-100), followed by centrifugation at 3000*g* and 4 °C for 10 min. A volume of 1 mL of cell lysis buffer [100 mM Tris–HCl, pH 7.9, at 4 °C, 1.5 mM NaCl, 1% (m/v) cetyltrimethylammonium bromide (CTAB)] was added to the pellet of the fifth washing step and the suspension was incubated in a water bath at 65 °C for 30 min. Four consecutive cycles of freezing the sample in liquid nitrogen and thawing it at 65 °C in a water bath were performed. After centrifugation at 3000*g* for 30 min at 4 °C, the supernatant was collected and washed three times with 1 volume of phenol/chloroform/isoamyl alcohol (25:24:1, v/v/v) each. After centrifuging for 15 min at 3000*g* and 4 °C, the aqueous layer was washed three times with 1 volume of chloroform each. Metagenomic DNA was precipitated by adding 1 volume 2-propanol and incubation on ice for 30 min and was collected by centrifugation at 3000*g* for 15 min at 4 °C; the resulting DNA pellet was washed by resuspension in 1 mL of 70% (v/v) ethanol (0 °C), followed by centrifugation at 3000*g* for 10 min. The washing step was repeated three times before the pellet was dried, dissolved in 300 μL Tris–EDTA (TE) buffer, and stored at −80 °C.

### Degenerated Primer Design

Full-length bacterial TYR sequences were obtained from the databases linked to the respective entry in the UniProtKB databank.^60^ Sequences from organisms indigenous to soil and/or peatlands were aligned using the Kalign Tool (http://msa.sbc.su.se/cgi-bin/msa.cgi),^88^ and degenerated primers were designed to bind to the regions showing the highest level of conservation (Figure 2). Desalted primers were obtained from a commercial supplier (Sigma-Aldrich, Vienna, Austria).

### Amplification and Cloning of Tyrosinase Partial Sequences, the Full-Length Tyrosinase Sequence, and the Codon-Optimized Caddie Protein

For the identification of the TYR partial sequences and the full-length *Sz*TYR sequence, PCR reactions were performed using 150 ng of metagenomic DNA extracted from peat samples as a template. Degenerated type III copper protein primers and MelC-specific primers equipped with the recognition site for *Sap*I (5′-GCTCTTC-3′) as well as the required 5′ overhangs (fwd: 5′-ATG-3′; rev: 5′-CCC-3′) (Table S1) were used at an annealing temperature of 68 °C using Q5 High-Fidelity DNA polymerase according to the PCR setup recommended by the supplier. Amplicons were cloned into the pENTRY-IBA51 vector carrying a kanamycin resistance gene. Plasmids containing putative tyrosinase partial sequences were transformed into *E. coli* TOP 10 cells (Thermo Fisher, Waltham), isolated from single colonies and sequenced in the forward and reverse direction by a commercial supplier (Microsynth GmbH, Vienna, Austria; Table S1).

For the construction of the expression vector, the sequence of the codon-optimized (toward the codon usage of *E. coli*) caddie protein (Table S6) obtained from Eurofins Genomics (Ebersberg, Germany) was introduced into the pGEX-6P-SG vector^89^ adjacent to the GST-tag using the restriction endonuclease *Esp*3I *via* the restriction enzyme recognition sites introduced into the optimized sequence. The sequence-verified ORF of full-length *Sz*TYR including a separate *tac*-promotor and a *lac*-operator was then cloned into the expression vector (already containing the sequence of the caddie protein) using two pairs of insertion primers (Table S1) and the restriction endonuclease *Esp*3I.

### Heterologous Expression and Purification of Recombinant *Sz*TYR

The pGEX-6P expression vector carrying the *Sz*TYR gene and the codon-optimized version of the caddie protein A0A2S3Y8X5 fused with a GST-tag, both under the control of separate *tac*-promotors and *lac*-operators, was transformed into *E. coli* BL21(DE3) cells. A volume of 100 mL of LB medium (10 g/L tryptone, 10 g/L NaCl, and 5 g/L yeast extract) was inoculated with a freshly transformed single colony and incubated at 37 °C and 230 rpm until an OD_600_ value of 0.8–1.0 was reached. Then, 0.5 mM isopropyl-β-d-thiogalactopyranoside (IPTG) and 0.5 mM CuSO_4_ were added, and the expression batch was incubated at 19 °C and 230 rpm for 60 h.

The expression medium was centrifuged for 10 min at 5000*g*, and the supernatant was precipitated by adding 3.12 g of (NH_4_)_2_SO_4_ (45% saturation) and incubating at 0 °C for 45 min. The pellet was collected by centrifugation at 45 000*g* for 15 min at 4 °C and resuspended in 10 mM Tris–HCl, pH 7.5 (at 4 °C). A Vivaspin ultrafiltration device (VWR, molecular weight cutoff of 30 kDa, 20 mL volume) was used to remove residual salts, and the sample was applied to a MonoQ anion exchange column (GE Healthcare, Freiburg, Germany) in 10 mM Tris–HCl, pH 7.5. Using 10 mM Tris–HCl, pH 7.5, as a starting buffer, the sample eluted without binding to the column (Figure S8). The purity of the sample was checked by 12.5% SDS-PAGE performed under denaturing conditions (Figure 3A).

The copper incorporation of *Sz*TYR was assessed photometrically using 2,2′-biquinoline according to the method published by Hanna et al.^90^

### Molecular Mass Determination of *Sz*TYR

Mass spectrometry was performed on an LTQ Orbitrap Velos mass spectrometer (Thermo Fisher Scientific, Bremen, Germany) equipped with a nanospray ion source (ion transfer capillary temperature: 300 °C; electrospray voltage: 2.1 kV). The sample was loaded on a trap column and separation was carried out on a C4 analytical column (50 cm × 75 μm Accucore C4, 2.6 μm, 150 Å from Thermo Fisher Scientific) at a flow rate of 300 nL/min. The mobile phase A comprised 2% acetonitrile, 98% H_2_O, and 0.1% formic acid. The mobile phase B comprised 80% acetonitrile, 20% H_2_O, and 0.1% formic acid.

### Biochemical Characterization of *Sz*TYR

Spectroscopic investigations of the formation of the oxygen-bound form of *Sz*TYR were performed on a Shimadzu UV-1800 spectrophotometer. A 0.5 g/L enzyme solution (16.2 μM) was mixed with increasing molar equivalents of H_2_O_2_ at 25 °C in 50 mM Tris–HCl, pH 9.0. Absorption spectra were recorded from 500 to 250 nm (Figure S9).

Since the sampling point of *Sz*TYR shows a pH value of 9.0, its pH profile (Figure 4) was investigated using 1.2 μg of enzyme and 1 mM tyramine as the substrate in a total volume of 200 μL in increments of 0.5 pH units ranging from pH 5.5 to 11.5. Buffer (sodium phosphate: pH 5.5–7.5; Tris–HCl: pH 7.5–9.5; *N*-cyclohexyl-3-aminopropanesulfonic acid (CAPS): pH 9.5–11.5) was added to a final concentration of 50 mM and measurements were performed on a TECAN infinity M200 photometer (Tecan, Salzburg, Austria) in 96-well plates in triplicate.

The temperature profile of *Sz*TYR was determined using 6 μg of enzyme and 1 mM tyramine as a substrate in a total volume of 1000 μL in increments of 5 °C ranging from 5 to 80 °C. Tris–HCl, pH 9.0 (adjusted to the respective temperature) was added to a final concentration of 50 mM. Measurements were performed on a Shimadzu UV-1800 spectrophotometer (Shimadzu Deutschland, Duisburg, Germany) attached to a circulation heater.

For the kinetic investigations, seven to eight different substrate molarities were mixed with adequate amounts of *Sz*TYR (Table S10) in 50 mM Tris–HCl pH 9.0, and initial reaction velocities were plotted into a Michaelis–Menten diagram. *v*_max_ and *K*_m_ values were calculated using nonlinear curve fitting (to the Michaelis–Menten equation) performed by OriginPro 8 software (Figure S13). All measurements were performed in triplicate.

For the substrate scope assay, 20 μg of *Sz*TYR was mixed with 1 mM substrate (*p*-coumaric acid, caffeic acid, protocatechuic acid, gallic acid, ferulic acid, vanillic acid, vanillin) and 50 mM Tris–HCl, pH 8.5 (to reduce auto-oxidation), in a total volume of 200 μL. The activity was assessed visually by a change in color (Figure S11).

## Supporting Information Available

Information concerning the development of a metagenomic DNA extraction protocol and the quality assessment of the DNA extract; a thermofluor assay of *Sz*TYR; investigation of the copolymerization of various phenolic substrates; sampling sites and their characteristics and 16S RNA analysis; tables containing primer sequences, sequence information, and sequence analysis of the identified partial sequences and 16S RNA sequences and biochemical data of *Sz*TYR (molecular mass, amounts of *Sz*TYR, wavelengths, and molar extinction coefficients used for kinetic measurements and melting temperatures); figures illustrating the composition of *Sz*TYR on a genetic and structural level, the quality of metagenomic DNA extracts, the diversity of bacterial TYRs present in peatlands, and the purification process and biochemical investigations of *Sz*TYR (PDF)

## References

[ref1] Maltby E.; Immirzi P. Carbon Dynamics in Peatlands and Other Wetland Soils Regional and Global Perspectives. Chemosphere 1993, 27, 999–1023. 10.1016/0045-6535(93)90065-D.

[ref2] Gorham E. Northern Peatlands: Role in the Carbon Cycle and Probable Responses to Climatic Warming. Ecol. Appl. 1991, 1, 182–195. 10.2307/1941811. 27755660

[ref3] Dean W. E.; Gorham E. Magnitude and Significance of Carbon Burial in Lakes, Reservoirs, and Peatlands. Geology 1998, 26, 535–538. 10.1130/0091-7613(1998)0262.3.CO;2.

[ref4] Oechel W. C.; Hastings S. J.; Vourlitis G.; Jenkins M.; Riechers G.; Grulke N. Recent Change of Arctic Tundra Ecosystems from a Net Carbon Dioxide Sink to a Source. Nature 1993, 361, 520–523. 10.1038/361520a0.

[ref5] Yu Z. C. Northern Peatland Carbon Stocks and Dynamics: A Review. Biogeosciences 2012, 9, 4071–4085. 10.5194/bg-9-4071-2012.

[ref6] Stevenson F. J. Humus Chemistry: Genesis, Composition, Reactions, 2nd ed.; Wiley: New York, 1994.

[ref7] Freeman C.; Fenner N.; Ostle N. J.; Kang H.; Dowrick D. J.; Reynolds B.; Lock M. A.; Sleep D.; Hughes S.; Hudson J. Export of Dissolved Organic Carbon from Peatlands under Elevated Carbon Dioxide Levels. Nature 2004, 430, 195–198. 10.1038/nature02707. 15241411

[ref8] Fenner N.; Freeman C.; Reynolds B. Hydrological Effects on the Diversity of Phenolic Degrading Bacteria in a Peatland: Implications for Carbon Cycling. Soil Biol. Biochem. 2005, 37, 1277–1287. 10.1016/j.soilbio.2004.11.024.

[ref9] Verhoeven J. T. A.; Liefveld W. M. The Ecological Significance of Organochemical Compounds in *Sphagnum*. Acta Bot. Neerl. 1997, 46, 117–130. 10.1111/plb.1997.46.2.117.

[ref10] Freeman C.; Ostle N.; Kang H. An Enzymic “latch” on a Global Carbon Store. Nature 2001, 409, 149 10.1038/35051650. 11196627

[ref11] Freeman C.; Ostle N. J.; Fenner N.; Kang H. A Regulatory Role for Phenol Oxidase during Decomposition in Peatlands. Soil Biol. Biochem. 2004, 36, 1663–1667. 10.1016/j.soilbio.2004.07.012.

[ref12] Bonnett S. A. F.; Ostle N.; Freeman C. Seasonal Variations in Decomposition Processes in a Valley-Bottom Riparian Peatland. Sci. Total Environ. 2006, 370, 561–573. 10.1016/j.scitotenv.2006.08.032. 17007907

[ref13] Sinsabaugh R. L. Phenol Oxidase, Peroxidase and Organic Matter Dynamics of Soil. Soil Biol. Biochem. 2010, 42, 391–404. 10.1016/j.soilbio.2009.10.014.

[ref14] Kim H.; Yeon Y. J.; Choi Y. R.; Song W.; Pack S. P.; Choi Y. S. A Cold-Adapted Tyrosinase with an Abnormally High Monophenolase/Diphenolase Activity Ratio Originating from the Marine Archaeon *Candidatus Nitrosopumilus koreensis*. Biotechnol. Lett. 2016, 38, 1535–1542. 10.1007/s10529-016-2125-0. 27193894

[ref15] Claus H.; Decker H. Bacterial Tyrosinases. Syst. Appl. Microbiol. 2006, 29, 3–14. 10.1016/j.syapm.2005.07.012. 16423650

[ref16] Pretzler M.; Bijelic A.; Rompel A. Fungal Tyrosinases: Why Mushrooms Turn Brown. In Elsevier Reference Module in Chemistry, Molecular Sciences and Chemical Engineering; Reedijk J., Ed.; Elsevier: Waltham, MA, 2015.

[ref17] Kaintz C.; Molitor C.; Thill J.; Kampatsikas I.; Michael C.; Halbwirth H.; Rompel A. Cloning and Functional Expression in *E. coli* of a Polyphenol Oxidase Transcript from *Coreopsis grandiflora* Involved in Aurone Formation. FEBS Lett. 2014, 588, 3417–3426. 10.1016/j.febslet.2014.07.034. 25109778 PMC4158910

[ref18] Kampatsikas I.; Bijelic A.; Pretzler M.; Rompel A. Three Recombinantly Expressed Apple Tyrosinases Suggest the Amino Acids Responsible for Mono- versus Diphenolase Activity in Plant Polyphenol Oxidases. Sci. Rep. 2017, 7, 8860 10.1038/s41598-017-08097-5. 28821733 PMC5562730

[ref19] Derardja A.; Pretzler M.; Kampatsikas I.; Barkat M.; Rompel A. Purification and Characterization of Latent Polyphenol Oxidase from Apricot (*Prunus armeniaca* L.). J. Agric. Food Chem. 2017, 65, 8203–8212. 10.1021/acs.jafc.7b03210. 28812349 PMC5609118

[ref20] Panis F.; Rompel A. Identification of the Amino Acid Position Controlling the Different Enzymatic Activities in Walnut Tyrosinase Isoenzymes (*Jr*PPO1 and *Jr*PPO2). Sci. Rep. 2020, 10, 10813 10.1038/s41598-020-67415-6. 32616720 PMC7331820

[ref21] Lai X.; Soler-Lopez M.; Wichers H. J.; Dijkstra B. W. Large-Scale Recombinant Expression and Purification of Human Tyrosinase Suitable for Structural Studies. PLoS One 2016, 11, e0161697 10.1371/journal.pone.0161697. 27551823 PMC4994950

[ref22] Kaintz C.; Mauracher S. G.; Rompel A. Type-3 Copper Proteins: Recent Advances on Polyphenol Oxidases. Adv. Protein. Chem. Struct. Biol. 2014, 97, 1–35. 10.1016/bs.apcsb.2014.07.001. 25458353

[ref23] Hearing J.; Tsukamoto K. Enzymatic Control of Pigmentation in Mammals. FASEB J. 1991, 5, 2902–2909. 10.1096/fasebj.5.14.1752358. 1752358

[ref24] Kanteev M.; Goldfeder M.; Fishman A. Structure-Function Correlations in Tyrosinases. Protein Sci. 2015, 24, 1360–1369. 10.1002/pro.2734. 26104241 PMC4570531

[ref25] Faccio G.; Kruus K.; Saloheimo M.; Thöny-Meyer L. Bacterial Tyrosinases and Their Applications. Process Biochem. 2012, 47, 1749–1760. 10.1016/j.procbio.2012.08.018.

[ref26] Eickman N. C.; Himmelwright R. S.; Solomon E. I. Geometric and Electronic Structure of Oxyhemocyanin: Spectral and Chemical Correlations to Met Apo, Half Met, Met, and Dimer Active Sites. Proc. Natl. Acad. Sci. U.S.A. 1979, 76, 2094–2098. 10.1073/pnas.76.5.2094. 287049 PMC383542

[ref27] Rompel A.; Fischer H.; Meiwes D.; Büldt-Karentzopoulos K.; Dillinger R.; Tuczek F.; Witzel H.; Krebs B. Purification and Spectroscopic Studies on Catechol Oxidases from *Lycopus europaeus* and *Populus nigra*: Evidence for a Dinuclear Copper Center of Type 3 and Spectroscopic Similarities to Tyrosinase and Hemocyanin. J. Biol. Inorg. Chem. 1999, 4, 56–63. 10.1007/s007750050289. 10499103

[ref28] Ramsden C. A.; Riley P. A. Tyrosinase: The Four Oxidation States of the Active Site and Their Relevance to Enzymatic Activation, Oxidation and Inactivation. Bioorg. Med. Chem. 2014, 22, 2388–2395. 10.1016/j.bmc.2014.02.048. 24656803

[ref29] Pretzler M.; Rompel A. What Causes the Different Functionality in Type-III-Copper Enzymes? A State of the Art Perspective. Inorg. Chim. Acta 2018, 481, 25–31. 10.1016/j.ica.2017.04.041.

[ref30] aBijelic A.; Pretzler M.; Molitor C.; Zekiri F.; Rompel A. The Structure of a Plant Tyrosinase from Walnut Leaves Reveals the Importance of “Substrate-Guiding Residues” for Enzymatic Specificity. Angew. Chem., Int. Ed. 2015, 54, 14677–14680. 10.1002/anie.201506994. PMC4678486 26473311

[ref31] Jackman M. P.; Hajnal A.; Lerch K. Albino Mutants of *Streptomyces glaucescens* Tyrosinase. Biochem. J. 1991, 274, 707–713. 10.1042/bj2740707. 1901488 PMC1149969

[ref32] Shuster Ben-Yosef V.; Sendovski M.; Fishman A. Directed Evolution of Tyrosinase for Enhanced Monophenolase/Diphenolase Activity Ratio. Enzyme Microb. Technol. 2010, 47, 372–376. 10.1016/j.enzmictec.2010.08.008.

[ref33] Goldfeder M.; Kanteev M.; Isaschar-Ovdat S.; Adir N.; Fishman A. Determination of Tyrosinase Substrate-Binding Modes Reveals Mechanistic Differences between Type-3 Copper Proteins. Nat. Commun. 2014, 5, 4505 10.1038/ncomms5505. 25074014

[ref34] Solem E.; Tuczek F.; Decker H. Tyrosinase versus Catechol Oxidase: One Asparagine Makes the Difference. Angew. Chem., Int. Ed. 2016, 55, 2884–2888. 10.1002/anie.201508534. 26773413

[ref35] Son H. F.; Lee S. H.; Lee S. H.; Kim H.; Hong H.; Lee U. J.; Lee P. G.; Kim B. G.; Kim K. J. Structural Basis for Highly Efficient Production of Catechol Derivatives at Acidic pH by Tyrosinase from *Burkholderia thailandensis*. ACS Catal. 2018, 8, 10375–10382. 10.1021/acscatal.8b02635.

[ref36] Kampatsikas I.; Bijelic A.; Rompel A. Biochemical and Structural Characterization of Tomato Polyphenol Oxidases Provide Novel Insights into Their Substrate Specificity. Sci. Rep. 2019, 9, 4022 10.1038/s41598-019-39687-0. 30858490 PMC6411738

[ref37] Panis F.; Kampatsikas I.; Bijelic A.; Rompel A. Conversion of Walnut Tyrosinase into a Catechol Oxidase by Site Directed Mutagenesis. Sci. Rep. 2020, 10, 1659 10.1038/s41598-020-57671-x. 32015350 PMC6997208

[ref38] Matoba Y.; Kumagai T.; Yamamoto A.; Yoshitsu H.; Sugiyama M. Crystallographic Evidence That the Dinuclear Copper Center of Tyrosinase Is Flexible during Catalysis. J. Biol. Chem. 2006, 281, 8981–8990. 10.1074/jbc.M509785200. 16436386

[ref39] Fenner N.; Freeman C. Drought-Induced Carbon Loss in Peatlands. Nat. Geosci. 2011, 4, 895–900. 10.1038/ngeo1323.

[ref40] Tveit A.; Schwacke R.; Svenning M. M.; Urich T. Organic Carbon Transformations in High-Arctic Peat Soils: Key Functions and Microorganisms. ISME J. 2013, 7, 299–311. 10.1038/ismej.2012.99. 22955232 PMC3554415

[ref41] Dimitriu P. A.; Lee D.; Grayston S. J. An Evaluation of the Functional Significance of Peat Microorganisms Using a Reciprocal Transplant Approach. Soil Biol. Biochem. 2010, 42, 65–71. 10.1016/j.soilbio.2009.10.001.

[ref42] Girkin N. T.; Lopes dos Santos R. A.; Vane C. H.; Ostle N.; Turner B. L.; Sjögersten S. Peat Properties, Dominant Vegetation Type and Microbial Community Structure in a Tropical Peatland. Wetlands 2020, 40, 1367–1377. 10.1007/s13157-020-01287-4.

[ref43] Tveit A. T.; Urich T.; Svenning M. M. Metatranscriptomic Analysis of Arctic Peat Soil Microbiota. Appl. Environ. Microbiol. 2014, 80, 5761–5772. 10.1128/AEM.01030-14. 25015892 PMC4178616

[ref44] Kotiaho M.; Fritze H.; Merilä P.; Tuomivirta T.; Väliranta M.; Korhola A.; Karofeld E.; Tuittila E. S. Actinobacteria Community Structure in the Peat Profile of Boreal Bogs Follows a Variation in the Microtopographical Gradient Similar to Vegetation. Plant Soil 2013, 369, 103–114. 10.1007/s11104-012-1546-3.

[ref45] Dedysh S. N.; Pankratov T. A.; Belova S. E.; Kulichevskaya I. S.; Liesack W. Phylogenetic Analysis and *In Situ* Identification of *Bacteria* Community Composition in an Acidic *Sphagnum* Peat Bog. Appl. Environ. Microbiol. 2006, 72, 2110–2117. 10.1128/AEM.72.3.2110-2117.2006. 16517660 PMC1393241

[ref46] Lewin G. R.; Carlos C.; Chevrette M. G.; Horn H. A.; McDonald B. R.; Stankey R. J.; Fox B. G.; Currie C. R. Evolution and Ecology of Actinobacteria and Their Bioenergy Applications. Annu. Rev. Microbiol. 2016, 70, 235–254. 10.1146/annurev-micro-102215-095748. 27607553 PMC5703056

[ref47] Leu W. M.; Chen L. Y.; Liaw L. L.; Lee Y. H. W. Secretion of the *Streptomyces* Tyrosinase Is Mediated through Its Trans-Activator Protein, MelC1. J. Biol. Chem. 1992, 267, 20108–20113. 10.1016/S0021-9258(19)88672-6. 1400329

[ref48] Chen L. Y.; Leu W. M.; Wang K. T.; Lee Y. H. W. Copper Transfer and Activation of the *Streptomyces* Apotyrosinase Are Mediated through a Complex Formation between Apotyrosinase and Its Trans- Activator MelC1. J. Biol. Chem. 1992, 267, 20100–20107. 10.1016/S0021-9258(19)88671-4. 1400328

[ref49] Ryu J.; Byun H.; Park J. P.; Park J.; Noh K. H.; Chung J. H.; Lee H.; Ahnb J. H. Tat-Dependent Heterologous Secretion of Recombinant Tyrosinase by *Pseudomonas fluorescens* is Aided by a Translationally Fused Caddie Protein. Appl. Environ. Microbiol. 2019, 85, e01350-19 10.1128/AEM.01350-19. 31399411 PMC6805089

[ref50] Matoba Y.; Kihara S.; Muraki Y.; Bando N.; Yoshitsu H.; Kuroda T.; Sakaguchi M.; Kayama K.; Tai H.; Hirota S.; Ogura T.; Sugiyama M. Activation Mechanism of the *Streptomyces* Tyrosinase Assisted by the Caddie Protein. Biochemistry 2017, 56, 5593–5603. 10.1021/acs.biochem.7b00635. 28902505

[ref51] Matoba Y.; Kihara S.; Bando N.; Yoshitsu H.; Sakaguchi M.; Kayama K.; Yanagisawa S.; Ogura T.; Sugiyama M. Catalytic Mechanism of the Tyrosinase Reaction toward the Tyr 98 Residue in the Caddie Protein. PLoS Biol. 2018, 16, e3000077 10.1371/journal.pbio.3000077. 30596633 PMC6312201

[ref52] Taylor P. D.; Toseland C. P.; Attwood T. K.; Flower D. R. TATPred: A Bayesian Method for the Identification of Twin Arginine Translocation Pathway Signal Sequences. Bioinformation 2006, 1, 184–187. 10.6026/97320630001184. 17597885 PMC1891679

[ref53] Schaerlaekens K.; Van Mellaert L.; Lammertyn E.; Geukens N.; Anné J. The Importance of the Tat-Dependent Protein Secretion Pathway in *Streptomyces* as Revealed by Phenotypic Changes in Tat Deletion Mutants and Genome Analysis. Microbiology 2004, 150, 21–31. 10.1099/mic.0.26684-0. 14702394

[ref54] Pundir S.; Martin M. J.; O’Donovan C. UniProt Tools. Curr. Protoc. Bioinf. 2016, 53, 1.29.1–1.29.15. 10.1002/0471250953.bi0129s53. PMC4941944 27010333

[ref55] Piñero S.; Rivera J.; Romero D.; Cevallos M. A.; Martínez A.; Bolívar F.; Gosset G. Tyrosinase from *Rhizobium etli* Is Involved in Nodulation Efficiency and Symbiosis-Associated Stress Resistance. J. Mol. Microbiol. Biotechnol. 2007, 13, 35–44. 10.1159/000103595. 17693711

[ref56] Cabrera-Valladares N.; Martínez A.; Piñero S.; Lagunas-Muñoz V. H.; Tinoco R.; De Anda R.; Vázquez-Duhalt R.; Bolívar F.; Gosset G. Expression of the MelA Gene from *Rhizobium etli* CFN42 in *Escherichia coli* and Characterization of the Encoded Tyrosinase. Enzyme Microb. Technol. 2006, 38, 772–779. 10.1016/j.enzmictec.2005.08.004.

[ref57] Sun H.; Terhonen E.; Koskinen K.; Paulin L.; Kasanen R.; Asiegbu F. O. Bacterial Diversity and Community Structure along Different Peat Soils in Boreal Forest. Appl. Soil Ecol. 2014, 74, 37–45. 10.1016/j.apsoil.2013.09.010.

[ref58] Manteca A.; Sanchez J. *Streptomyces* Development in Colonies and Soils. Appl. Environ. Microbiol. 2009, 75, 2920–2924. 10.1128/AEM.02288-08. 19270137 PMC2681692

[ref59] Hruska K.; Kaevska M. Mycobacteria in Water, Soil, Plants and Air: A Review. Vet. Med. 2013, 57, 623–679. 10.17221/6558-VETMED.

[ref60] UniProt: The Universal Protein Knowledgebase in 2021. Nucleic Acids Res. 2021, 49, D480–D489. 10.1093/nar/gkaa1100. 33237286 PMC7778908

[ref61] Ikeda K.; Masujima T.; Suzuki K.; Sugiyama M. Cloning and Sequence Analysis of the Highly Expressed Melanin-Synthesizing Gene Operon from *Streptomyces castaneoglobisporus*. Appl. Microbiol. Biotechnol. 1996, 45, 80–85. 10.1007/s002530050652. 8920182

[ref62] Wang G.; Aazaz A.; Peng Z.; Shen P. Cloning and Overexpression of a Tyrosinase Gene Mel from *Pseudomonas maltophila*. FEMS Microbiol. Lett. 2000, 185, 23–27. 10.1111/j.1574-6968.2000.tb09035.x. 10731602

[ref63] Lee N.; Lee S. H.; Baek K.; Kim B. G. Heterologous Expression of Tyrosinase (MelC2) from *Streptomyces avermitilis* MA4680 in *E. coli* and Its Application for Ortho-Hydroxylation of Resveratrol to Produce Piceatannol. Appl. Microbiol. Biotechnol. 2015, 99, 7915–7924. 10.1007/s00253-015-6691-1. 26036705

[ref64] Li Y.; Li Y.; Li Q.; Gao J.; Wang J.; Luo Y.; Fan X.; Gu P. Biosynthetic and Antimicrobial Potential of Actinobacteria Isolated from Bulrush Rhizospheres Habitat in Zhalong Wetland, China. Arch. Microbiol. 2018, 200, 695–705. 10.1007/s00203-018-1474-6. 29368168

[ref65] Matoba Y.; Bando N.; Oda K.; Noda M.; Higashikawa F.; Kumagai T.; Sugiyama M. A Molecular Mechanism for Copper Transportation to Tyrosinase That Is Assisted by a Metallochaperone, Caddie Protein. J. Biol. Chem. 2011, 286, 30219–30231. 10.1074/jbc.M111.256818. 21730070 PMC3191061

[ref66] Kanteev M.; Goldfeder M.; Chojnacki M.; Adir N.; Fishman A. The Mechanism of Copper Uptake by Tyrosinase from *Bacillus megaterium*. J. Biol. Inorg. Chem. 2013, 18, 895–903. 10.1007/s00775-013-1034-0. 24061559

[ref67] Kampatsikas I.; Rompel A. Similar but Still Different – Which Amino Acid Residues Are Responsible for Varying Activities in Type-III Copper Enzymes?. ChemBioChem 2021, 22, 1161–1175. 10.1002/cbic.202000647. 33108057 PMC8049008

[ref68] Kim M.; Oh H. S.; Park S. C.; Chun J. Towards a Taxonomic Coherence between Average Nucleotide Identity and 16S rRNA Gene Sequence Similarity for Species Demarcation of Prokaryotes. Int. J. Syst. Evol. Microbiol. 2014, 64, 346–351. 10.1099/ijs.0.059774-0. 24505072

[ref69] Kohashi P. Y.; Kumagai T.; Matoba Y.; Yamamoto A.; Maruyama M.; Sugiyama M. An Efficient Method for the Overexpression and Purification of Active Tyrosinase from *Streptomyces castaneoglobisporus*. Protein Expression Purif. 2004, 34, 202–207. 10.1016/j.pep.2003.11.015. 15003252

[ref70] Goldfeder M.; Kanteev M.; Adir N.; Fishman A. Influencing the Monophenolase/Diphenolase Activity Ratio in Tyrosinase. Biochim. Biophys. Acta, Proteins Proteomics 2013, 1834, 629–633. 10.1016/j.bbapap.2012.12.021. 23305929

[ref71] Harper S.; Speicher D. W. Purification of Proteins Fused to Glutathione S-Tranferase. Methods Mol. Biol. 2011, 681, 259–280. 10.1007/978-1-60761-913-0_14. 20978970 PMC3584333

[ref72] Costa S.; Almeida A.; Castro A.; Domingues L. Fusion Tags for Protein Solubility, Purification, and Immunogenicity in *Escherichia coli*: The Novel Fh8 System. Front. Microbiol. 2014, 5, 63 10.3389/fmicb.2014.00063. 24600443 PMC3928792

[ref73] Ito M.; Oda K. An Organic Solvent Resistant Tyrosinase from *Streptomyces* sp. REN-21: Purification and Characterization. Biosci., Biotechnol., Biochem. 2000, 64, 261–267. 10.1271/bbb.64.261. 10737179

[ref74] Bubacco L.; Vijgenboom E.; Gobin C.; Tepper A. W. J. W.; Salgado J.; Canters G. W. Kinetic and Paramagnetic NMR Investigations of the Inhibition of *Streptomyces antibioticus* Tyrosinase. J. Mol. Catal. B: Enzym. 2000, 8, 27–35. 10.1016/S1381-1177(99)00064-8.

[ref75] Dolashki A.; Gushterova A.; Voelter W.; Tchorbanov B. Purification and Characterization of Tyrosinases from *Streptomyces albus*. Z. Naturforsch., C: J. Biosci. 2009, 64, 724–732. 10.1515/znc-2009-9-1019. 19957443

[ref76] Liao Y.-D.; Jeng J.-C.; Wang C.-F.; Wang S.-C.; Chang S.-T. Removal of N-Terminal Methionine from Recombinant Proteins by Engineered *E. coli* Methionine Aminopeptidase. Protein Sci. 2004, 13, 1802–1810. 10.1110/ps.04679104. 15215523 PMC2279930

[ref77] Wingfield P. T. N-Terminal Methionine Processing. Curr. Protoc. Protein Sci. 2017, 88, 6.14.1–6.14.3. 10.1002/cpps.29. 28369664 PMC5663234

[ref78] Zekiri F.; Molitor C.; Mauracher S. G.; Michael C.; Mayer R. L.; Gerner C.; Rompel A. Purification and Characterization of Tyrosinase from Walnut Leaves (*Juglans regia*). Phytochemistry 2014, 101, 5–15. 10.1016/j.phytochem.2014.02.010. 24613318 PMC3989047

[ref79] Eicken C.; Zippel F.; Büldt-Karentzopoulos K.; Krebs B. Biochemical and Spectroscopic Characterization of Catechol Oxidase from Sweet Potatoes (*Ipomoea batatas*) Containing a Type-3 Dicopper Center. FEBS Lett. 1998, 436, 293–299. 10.1016/S0014-5793(98)01113-2. 9781698

[ref80] Rompel A.; Büldt-Karentzopoulos K.; Molitor C.; Krebs B. Purification and Spectroscopic Studies on Catechol Oxidase from Lemon Balm (*Melissa officinalis*). Phytochemistry 2012, 81, 19–23. 10.1016/j.phytochem.2012.05.022. 22727580

[ref81] Umek N.; Geršak B.; Vintar N.; Šoštarič M.; Mavri J. Dopamine Autoxidation Is Controlled by Acidic pH. Front. Mol. Neurosci. 2018, 11, 467 10.3389/fnmol.2018.00467. 30618616 PMC6305604

[ref82] Liu N.; Zhang T.; Wang Y. J.; Huang Y. P.; Ou J. H.; Shen P. A Heat Inducible Tyrosinase with Distinct Properties from *Bacillus thuringiensis*. Lett. Appl. Microbiol. 2004, 39, 407–412. 10.1111/j.1472-765X.2004.01599.x. 15482430

[ref83] Nikolaivits E.; Dimarogona M.; Karagiannaki I.; Chalima A.; Fishman A.; Topakas E. Versatile Fungal Polyphenol Oxidase with Chlorophenol Bioremediation Potential: Characterization and Protein Engineering. Appl. Environ. Microbiol. 2018, 84, e01628-18 10.1128/AEM.01628-18. 30266731 PMC6238066

[ref84] Krachler R.; Krachler R.; Gülce F.; Keppler B. K.; Wallner G. Uranium Concentrations in Sediment Pore Waters of Lake Neusiedl, Austria. Sci. Total Environ. 2018, 633, 981–988. 10.1016/j.scitotenv.2018.03.259. 29758919

[ref85] Fussmann D.; Von Hoyningen-Huene A. J. E.; Reimer A.; Schneider D.; Babková H.; Peticzka R.; Maier A.; Arp G.; Daniel R.; Meister P. Authigenic Formation of Ca-Mg Carbonates in the Shallow Alkaline Lake Neusiedl, Austria. Biogeosciences 2020, 17, 2085–2106. 10.5194/bg-17-2085-2020.

[ref86] Krachler R.; von der Kammer F.; Jirsa F.; Süphandag A.; Krachler R. F.; Plessl C.; Vogt M.; Keppler B. K.; Hofmann T. Nanoscale Lignin Particles as Sources of Dissolved Iron to the Ocean. Global Biogeochem. Cycles 2012, 26, GB3024 10.1029/2012GB004294.

[ref87] Tarnawski M.; Depta K.; Grejciun D.; Szelepin B. HPLC Determination of Phenolic Acids and Antioxidant Activity in Concentrated Peat Extract - A Natural Immunomodulator. J. Pharm. Biomed. Anal. 2006, 41, 182–188. 10.1016/j.jpba.2005.11.012. 16368219

[ref88] Lassmann T.; Sonnhammer E. L. L. Kalign--an accurate and fast multiple sequence alignment algorithm. BMC Bioinf. 2005, 6, 298 10.1186/1471-2105-6-298. PMC1325270 16343337

[ref89] Biundo A.; Braunschmid V.; Pretzler M.; Kampatsikas I.; Darnhofer B.; Birner-Gruenberger R.; Rompel A.; Ribitsch D.; Guebitz G. M. Polyphenol Oxidases Exhibit Promiscuous Proteolytic Activity. Commun. Chem. 2020, 3, 62 10.1038/s42004-020-0305-2. PMC9814219 36703476

[ref90] Hanna P. M.; Tamilarasan R.; McMillin D. R. Cu(I) Analysis of Blue Copper Proteins. Biochem. J. 1988, 256, 1001–1004. 10.1042/bj2561001. 3223941 PMC1135515

