# Supplementary material for: Expression, Purification, and Characterization of a Well-Adapted Tyrosinase from Peatlands Identified by Partial Community Analysis
Source: Environ Sci Technol. 2021 Jun 22;55(16):11445–54. doi: 10.1021/acs.est.1c02514 (PMC8375020; doi:10.1021/acs.est.1c02514)
Supplement: Supplementary file 1 — es1c02514_si_001.pdf [file es1c02514_si_001.pdf]

Supporting Information

Expression, purification, and characterization of a well-adapted tyrosinase from peatlands identified by partial community analysis.

Felix Panis [1], Rudolf F. Krachler [2], Regina Krachler [2], Annette Rompel *[1].

1 Universität Wien, Fakultät für Chemie, Institut für Biophysikalische Chemie, Althanstraße 14, 1090 Wien, Austria; https://www.bpc.univie.ac.at

*Correspondence to: annette.rompel@univie.ac.at

2 Universität Wien, Fakultät für Chemie, Institut für Anorganische Chemie, Althanstraße 14, 1090 Wien.

33 Pages

10 Tables

13 Figures

Content

1. Supporting data.. S4

Development of a metagenomic DNA extraction protocol and quality assessment of the DNA extract.. S4

Thermofluor assay of *Sz*TYR.. S4

Investigation of the co-polymerization of various phenolic substrates S5

Sampling sites and their characteristics.. S5

2. Materials and methods.. S7

Quality assessment of the metagenomic DNA extract S7

Thermofluor assay of *Sz*TYR.. S7

BLAST search, alignment, and phylogenetic tree construction S7

16S RNA analysis. S7

Investigation of the co-polymerization of various phenolic substrates S8

3. Tables.. S9

Table S1. List of primers.. S9

Table S2. Partial TYR nucleotide sequences S12

Table S3. High identity matches (> 75 %) identified by a BLAST search S13

Table S4. Partial TYR amino acid sequences S14

Table S5. Identity matrix of the 19 identified TYR partial amino acid sequences S14

Table S6. Full-length sequences of MelC2 (*Sz*TYR), MelC1 (caddie protein) and MelC1 codon-optimized (caddie protein).. S15

Table S7. 16S RNA sequence S16

Table S8. Calculated and measured molecular mass of recombinantly expressed *Sz*TYR. S17

Table S9. Melting temperatures (T_m values) of *Sz*TYR S17

Table S10. Amounts of *Sz*TYR (µg), wavelengths (λ), and molar extinction coefficients (ε) used for kinetic measurements.. S**Error! Bookmark not defined.**

4. Figures.. S19

Figure S1. Active center of a bacterial TYR with tyrosine in the active site S19

Figure S2. Localization of MelC1 (caddie protein, blue) and MelC2 (tyrosinase, green) within the MelC operon from *Streptomyces* sp.. S20

Figure S3. Sequence covered by the identified TYR partial sequences.................................... S21

Figure S4. Metagenomic DNA extracted from soil samples.. S22

Figure S5. Phylogenetic tree of the 19 identified nucleotide sequences S23

Figure S6. Multiple sequence alignment of a segment of the 19 partial TYR sequences (Table S4) around the activity controller residues.. S24

Figure S7. Structures of phenolic substrates..S25

Figure S8. Chromatogram of the purification of *Sz*TYR via anion exchange chromatography S26

Figure S9. UV-Vis absorption spectrum of a 0.5 g/l solution of *Sz*TYR...................................... S27
Figure S10. Thermofluor assay of *Sz*TYR at different pH values. S28
Figure S11. Substrate scope assay of *Sz*TYR using phenolic compounds naturally abundant in peatlands S28
Figure S12. Co-polymerization of various phenolic substrates S29
Figure S13. Non-liner curve fitting of data points measured for *Sz*TYR during kinetic assays. S31
5. References.. S32

1. Supporting data

Development of a metagenomic DNA extraction protocol and quality assessment of the DNA extract. Prior to cell lysis and the extraction of DNA, the samples were washed with a humic substance removal solution adjusted to pH 9.0 (see Materials and methods) to efficiently remove fulvic acids and low molecular weight humic acids. Polyvinylpyrrolidone (PVP), which forms hydrogen bonds with phenolic compounds[1], was added to the humic substance removal solution, resulting in an increased efficiency of phenolic compound removal (compared to humic substance removal solution without PVP). The washed peat samples were subjected to different forms of cetyltrimethylammonium bromide (CTAB) assisted cell lysis, namely cell lysis *via* Ultra-Turrax, glass beads, and repetitive freeze-thaw cycles (Figure S4). Freezing samples in liquid nitrogen followed by incubation at 65 °C proved most efficient in terms of quality and quantity of the metagenomic DNA extract (Figure S4). To further minimize the co-extraction of humic substances a cell lysis buffer adjusted to pH 7.9 was used. At pH 7.9 high molecular weight humic substances (humins and high molecular weight humic acids) remain insoluble while low molecular weight humic substances (fulvic acids and low-molecular-weight humic acids) have been removed previously by the humic substance removal solution (pH 9.0) [2].
The purification of the crude metagenomic DNA extract was performed by phenol:chloroform:isoamyl alcohol extraction. Phenol, which is partially soluble in the aqueous phase and inhibits downstream applicability of the extracted metagenomic DNA (e.g. for PCR), was removed by extracting the aqueous layer with chloroform prior to the precipitation of DNA.

Co-extraction of humic substances is a major concern in the extraction of DNA from soils showing high organic matter content since they impede downstream applications[3,4]. Thus, the metagenomic DNA extracts were assessed in terms of PCR inhibition potential using *Taq*-polymerase and Q5 High-Fidelity DNA polymerase (see supporting Materials and methods). The DNA extracts showed limited DNA inhibition potential with Q5 High-Fidelity DNA polymerase tolerating higher DNA extract concentrations (corresponding to 150 ng metagenomic DNA per 10 µl PCR reaction) compared to *Taq*-polymerase (corresponding to 30 ng metagenomic DNA per 10 µl PCR reaction). Thus, all further PCR experiments were performed using Q5 High-Fidelity DNA polymerase.

Thermofluor assay of *Sz*TYR. To test the thermal stability of *Sz*TYR a thermofluor assay was performed. Since its sampling site exhibits a pH value of 9.0 – 9.5, *Sz*TYR (which shows a pH optimum of 9.0) was incubated at pH 7.0 – 11.0 (pH 7.0 – 9.0: TRIS-HCl; pH 9.0 – 11.0: CAPS) in increments of one pH unit (see supporting Materials and methods) and revealed a high level of thermal stability as unfolding occurred around 67 °C (at pH 9.0) (Figure S10). The pH value

showed only a limited influence on the thermal stability of *Sz*TYR, as T_m values ranged from 65.6 °C (pH 11.0) to 68.2 °C (pH 8.0). Fluorescence intensities reached a maximum (which indicates a high level of denaturation) at 75 – 80 °C, which is in accordance with the temperature profile measured for *Sz*TYR (Figure 4).

Investigation of the co-polymerization of various phenolic substrates. The co-polymerization of various phenolic compounds present in peatlands represents an environmentally important step in the long-time storage of organic carbon. As demonstrated in this study, *Sz*TYR displays the potential of efficiently converting monophenolic, diphenolic, and triphenolic compounds present in peatlands into the corresponding *o*-quinones. These *o*-quinones form high molecular weight polymers (melanins[5], fulvic acids, humic acids, and humins[6]) *via* a diverse set of spontaneous, non-enzymatic reactions, including nucleophilic Michael-1,6-addition, redox exchange, and intramolecular cyclization[7]. Thereby, the concentration of phenolic compounds is reduced, which potentially boosts the activity of soil organic matter degrading enzymes. To experimentally prove the co-polymerization of phenolic compounds naturally present in peatlands[8,9] a solution containing coumaric acid, caffeic acid, protocatechuic acid, and gallic acid (in the following referred to as "phenol-mix"; see supporting Materials and methods, Figure S7) was incubated with *Sz*TYR (in 50 mM TRIS- HCl pH 8.5). After 72 hours a dark precipitate indicated the formation of insoluble, high molecular weight polymers (Figure S12), which were separated from the supernatant by centrifugation. The phenolic content of the supernatant (phenol-mix after incubation with *Sz*TYR without insoluble, high molecular weight polymers) was determined using the folin-ciocalteu method[10] and revealed that after incubation with *Sz*TYR for 72 hours, the phenol content has been reduced by 55 % (compared to the phenol content of the phenol-mix prior to the addition of *Sz*TYR). Thus, the results prove the formation of high-molecular weight polymers from a complex mixture of phenolic compounds naturally present in peatlands.

Sampling sites and their characteristics. In order to get an orientation on the role of bacterial TYRs in peatlands, samples from two different types of peatlands (i.e. low pH raised bog and high pH salt marsh) were collected at the following locations: 48°30'18.0"N 14°51'43.3"E (Tanner Moor, an acidic raised bog in Upper Austria, Austria, September 2017) and 47°45'13.2"N 16°44'55.5"E (a soda-rich inland salt marsh in the riparian zone of Lake Neusiedl, Burgenland, Austria, July 2020). At both sites, samples were collected under wet conditions at 0 – 10 cm depth. The top layer (0 to 10 cm depth) contains the youngest peat which is mostly under aerobic conditions. Below 20 cm depth stability of the catotelm begins. The zone in

between (5 – 20 cm) is where water table fluctuation occurs. Peat samples were either processed within a few hours or shock-frozen in liquid nitrogen and stored at -80 °C.

2. Materials and methods

Quality assessment of the metagenomic DNA extract. The quantity and quality of the metagenomic DNA extract were assessed by 0.6 % (m/v) agarose gel electrophoresis (Fig. S4) stained with SYBR-Safe DNA gel stain (Thermo Fisher, Waltham, USA). The PCR-inhibition potential of the extract was assessed by adding 2 µl of the metagenomic DNA extract, adequately diluted with 1xTE buffer, to a 20 µl PCR reaction containing *Taq* DNA polymerase (NEB) or Q5 High-Fidelity DNA polymerase (NEB) using the PCR setup recommended by the supplier as well as the pENTRY-IBA51 vector and suitable primers (Table S1). Dilutions showing a clear band at the expected size (731 bp) were assessed as exhibiting a low PCR-inhibition potential.

Thermofluor assay of *Sz*TYR. 12.5 µM *Sz*TYR was mixed with 15 x SYPRO Orange Protein Gel Stain (Thermo Fisher, Waltham, USA) in 50 mM buffer (TRIS – HCL: pH 7.0, 8.0, 9.0; CAPS: pH 9.0, 10.0, 11.0) in a total volume of 100 µl. Fluorescence intensities were measured at 550 nm in increments of 1 °C from 10 – 98 °C. The melting temperatures (T_m values) were determined by calculating the midpoints of the sigmoidal parts of the intensity curves *via* non-linear curve fitting to the Boltzmann equation[11] as implemented in the OriginPro 8 software. Measurements were performed in triplicates on an Eppendorf Realplex^2 Mastercycler.

BLAST search, alignment, and phylogenetic tree construction. A BLAST search (https://www.uniprot.org/blast/) of the identified sequences (primer binding regions were excluded) against the *UniProtKB reference proteomes plus Swiss-Prot* databank was performed. Amino acid sequence alignments were performed using the Clustal Omega program implemented in the UniProt alignment tool (https://www.uniprot.org/align/)[12]. The BLOSUM-62 substitution matrix was used[13], the maximum E-score threshold was set to 10^{-4}, no filtering was applied and gaps were allowed.

Nucleotide alignments by multiple sequence comparison by log-expectation and the construction of a maximum likelihood phylogenetic tree were performed by the MEGA X software package[14]. The phylogenetic tree was edited using the FigTree v1.4.4 software (http://tree.bio.ed.ac.uk/software/figtree/). The Tamura 3 parameter model was selected as the substitution type applying gamma distribution with invariant sites.

16S RNA analysis. 16S RNA sequences were amplified from metagenomic DNA extracted from peat samples using 16S RNA standard primers suitable for cloning into the pENTRY-

IBA51 vector (Table S1). Q5 High-Fidelity DNA polymerase (NEB, Ipswich, USA) was used according to the PCR setup recommended by the supplier with 150 ng metagenomic DNA and 25 % (v/v) high GC enhancer (NEB, Ipswich, USA) added to the PCR reaction. Amplicons were cloned into the pENTRY-IBA51 vector using SapI as the restriction endonuclease. After cloning into *E. coli* TOP 10 cells (Thermo Fisher, Waltham, USA) single colonies were selected, plasmids were isolated and analyzed by Sanger sequencing.

Investigation of the co-polymerization of various phenolic substrates. To test the co-polymerization of various phenolic compounds a solution containing 2 mM coumaric acid, 2 mM caffeic acid, 2 mM protocatechuic acid, and 2 mM gallic acid was incubated with 20 µg *Sz*TYR in 50 mM TRIS – HCl (pH 8.5; to reduce autooxidation[15]) in a total volume of 200 µl. The mixture was incubated for 72 hours at room temperature.

For the determination of the total phenolic content 100 µg *Sz*TYR were added to 1 ml of a solution containing 2 mM coumaric acid, 2 mM caffeic acid, 2 mM protocatechuic acid, and 2 mM gallic acid (Figure S7) in 50 mM TRIS – HCl (pH 8.5). After incubation at room temperature for 72 hours, the mixture was centrifuged at 20,000 g for 10 minutes. The supernatant was separated from the pellet. To determine the phenolic content of the supernatant, 100 µl of the supernatant were mixed with 200 µl 10 % folin-ciocalteu reagent (Merck, Darmstadt, Germany) and 800 µl of an aqueous 700 mM Na_2CO_3 solution. The mixture was incubated at room temperature for 2 hours and the absorption was then measured on a Shimadzu UV-1800 spectrophotometer at 765 nm[10]. As a reference, the phenolic content of a solution containing 2 mM coumaric acid, 2 mM caffeic acid, 2 mM protocatechuic acid, and 2 mM gallic acid (without *Sz*TYR added) in 50 mM TRIS – HCl (pH 8.5) was determined according to the protocol described above. Photometric measurements were performed in triplicates.

3. Tables

fwd	5’-TTCCTGSCSTGGCACCG-3’
rev	5’-GCAGCCAGAASAYSGGRTC-3’
fwd cloning	5’-AGCGgctcttcaATGTTCCTGSCSTGGCACCG-3’
rev cloning	5’-AGCGgctcttctCCCGCAGCCAGAASAYSGGRTC-3’
sequencing primer pENTRY-IBA51 fwd	5’-ATAGGGGTTCCGCGCACATTTC-3’
sequencing primer pENTRY-IBA51 rev	5’-CCCCTGATTCTGTGGATAACCG-3’
fwd MelC	5’-AGCGgctcttcaaTGTCTCGAATCACCCGTCG-3’
rev MelC	5’-AGCGgctcttcaCCCCTACGCCATCGTGTCGTACG-3’
fwd TYR insertion primer 1	5’-ATCTTGcgtctcaTTGACAATTAATCATCGGCTCGTATAATGTGTGGAATTGTGAGCGGATAACAATTTCACACAGGAAACAGTATTCATGACCGTACGCAAGAACCAGG-3’
rev TYR insertion primer 1	5’-ATCTTGcgtctcaCTACGCCATCGTGTCGTACGTG-3’
fwd TYR insertion primer 2	5’-ATCTTGcgtctcaGGGAATAACCGTATTACCGCCTTTGAGTGAG-3’
rev TYR insertion primer 2	5’-ATCTTGcgtctctCATTCCACAGAATCAGGGGATAACGC-3’
fwd PCR inhibition potential	5’-CACGACAGGTTTCCCGACT-3’
rev PCR inhibition potential	5’-CCCCTGATTCTGTGGATAACCG-3’
16S RNA primer fwd	5’-AGCGgctcttcaATGAGAGTTTGATCMTGGCTCAG-3’
16S RNA primer rev	5’-AGCGgctcttctCCCCGGTTACCTTGTTACGACTT-3’

Table S1. List of primers. Degenerated type III copper protein primers (fwd, rev) and cloning primers for TYR partial sequences (fwd cloning, rev cloning). Primers used for sequencing sequences inserted into the pENTRY IBA51 vector. Primers used to obtain the sequence of the MelC operon of *Streptomyces sp.* ZL-24 containing A0A2S3Y8X5 and A0A2S3Y8X7 (fwd MelC, rev MelC) with suitable restriction enzyme recognition sites for cloning into the pENTRY-IBA51 vector. Primers used for the insertion of an Esp3I recognition site at the 3’ and 5’ ends of the TYR sequence (*Sz*TYR) (fwd TYR insertion primer 1, rev TYR insertion primer 1) and primers used for the insertion of Esp3I recognition sites into the pGEX-6P-SG vector (fwd TYR

insertion primer 2, rev TYR insertion primer 2). Primers used for the determination of the PCR inhibition potential of the metagenomic DNA extract (fwd PCR inhibition potential, rev PCR inhibition potential). Primers used for 16S RNA analysis experiments. The restriction enzyme recognition sites are indicated by small letters.

sequence 1	5’-GGCCTATCTCTACTACTTCGAGCGCATCTTGCGCTGGGCGGCGAACGATC<br>CGACGCTGGCGTTGCCCTACTGGAACTACCACGACGAGAACCAACGCACCA<br>TCCCTCAGGCTTACCGCGATGCGACGTTCGGCCAAGACAAAGCCCCCAACC<br>CGCTGTACCTGTCCGCCAACGCCCGCTTCACCGACAAGGATGGCAAGCCGC<br>AGCTGTTCCCAATGCGCGACGCCGACCTCAACCAGGGCTTGACCCAACTCG<br>CCGCCCCCTACGTCAGCACCGACGCCCTGCTGGCGACCGCGTTCACTGCCT<br>CGCCCCCCGCGCCGGTCAACACCACTTTCGGAAGCGCGTGGGCGTGTGAC<br>CAGACCTGTGCGTGCGCCGGAGGCGCGTTGGAGCGCATCCCGCACAACGC<br>CGTCCACAATGCCATCGGCGGCGCCCTGGTCACGACCGGCGGCAGTTTCTG<br>GGTCGGGTTCATGGGCGACATAACCACGTCGGCTCG-3’
sequence 2	5’-GATGTACGTTCTGAACTTTGAACGGATCGTCGCCAGGCACGTCGAGAGCC<br>TCGGCGGCCCTGCCGACTGGGCGTTGCCTTACTGGAATTACACGACCACGA<br>ATCCCGTCACCCTCGCGCTCCCGCCGGCGTTCCGCAGCCCGCTACTCCCGA<br>CCGGCGGCCCGAATCCACTCTACGTGGCGCTGCGGAACCCGGTGGCGAAC<br>GCGGGCAGCCCTGTTCTGGGTCGCCGCGACGTTTCGCTCAACTGCCTGAGT<br>ACGAGCGGGACCACCAGGCCGGGAGACTTTTTCGGCGGGTCACCCCCCGA<br>TCATAATGGCAGTCTGCCAGGGGCGCTGGAACTGACGCCGCACAATGCGAT<br>CCACAACCAGGTCGGTAAAACACCGGGAGGGTGGATGCAAGACCCTGACCT<br>GGCCGCACG-3’
sequence 3	5’-AATGTTTCTTTGCTATTTTGAGCTGCTCATTCGGCAGGCCTCTGGGCGCCC<br>AGACTTTACGCTGCCTTATTGGGACTACACCAACGCCAAGCACCCGGAATCT<br>GCGGCCTTGCCCGATGAATTTCACTGGCCCGGAGTGGTGTCAAACTCGCTTT<br>ACCGAGTAAACCGAAACAGGAAGGCCAATGCCGGCAAGCCGATTGACACGA<br>TCGGTCCGACCGGGCTAAACCTCGGTTCGCTTTCTGAGCGAACCTATCGCG<br>ACCATGGTGCGGCGATGGGCTTTGGCCAGAACCTGGACGTGTATCTGCACT<br>CCGACGTGCACATTCTCACCGGCAATTCGTTGGGAATGGGTTCGATTCCGTG<br>GGCTGCGAG-3’
sequence 4	5’-CGGTTATCTGATCGCTTTTGAGAAGGTGATCCGCGCGGCGGTGGTGGCC<br>GCGGGCGGTCCGGCCGACTGGGCCCTGCCCTACTGGAACTACTTCAAGCCA<br>AACCAGAACCAGCTGCCGCCGGCGTTCGCCACGCCGGACTGGCCAGACGG<br>CGCCGGCGACAACCCTCTGTATATCAAGCAACGCCACGGGCCGCGGAACAA<br>CGGCAACGTCTATGTGCTGTTGAGCAAAGTGAACCTGAAGGCGCTAGGCGA<br>CAACAATTTCATCGGCGCCGGAACCGGCGGCAGCCTGGGCTTCGGCGGCG<br>TGGTGACTGGATTCAGCCATCAGCACAGCCCGCATGGCGGCATCGAATCGC<br>AGCCCCATGACCAGGTGCACGGGCTGGTGGGCGGCGCCGACCCTACCACT<br>GGCCTGCCGGGCCTGATGTCCGACCCTGACACCGCCGGGCT-3’
sequence 5	5’-AATGTACGTTTTGAACTTTGAACGAATCGTCGCCATGCACGTGGCGAGTCT<br>CGGCGGCCCTGCCGACTGGGCGTTGCCGTACTGGAATTACACGACCACGAA<br>TCCCGCCACTCTTGAGCTTCCGCCGGCATTCCGCGACCCGGTACTCGCGAC<br>CGGGGCGCCGAATCACCTCTACGTAGCACTGCGAAACCCGTTGGCGAACGC<br>GGGTGGCGCTGTTCTGGGGCCCCGCGACGTTGCGCTCAACTGTCTGAGCG<br>CGAGCGGGACCACCATTCCGGGTGGTTTTTTCGGCGGCGCCCCACCTGATC<br>ATTCTGGTCATCTGGGTGGAGCGCTGGAATTGACACCCCATAATGCGATTCA<br>CAACCAAGTCGGTAAGACACTGGGTGGGTTGATGCAAGATCCCGACTTAGCA<br>GCACT-3’
sequence 6	5’-AATGTACATTTTGAATTTCGAACGCATCGTCGCCCGGCACGTGGTGAGTCT<br>CGGTGGCCCTGCGGATTGGGCGTTGCCGTACTGGAATTACACGACTTCGGA<br>CACAGCCACCCTGGCACTCCCTCCGGCATTCCGCAATCCGGCACTCCCGGG<br>CGGGGCGCCGAACCCACTCCACGTGGCCCTGCGAAACCCGGTGGCGAACG<br>CAGGCGGCTCTGTTCTGGGCCCCCACGATGTTGATCTCACCTGTCTCAATGC<br>AAGTGGAACCACTATTCCGGGTGGCTTTTTCGGCGGTGCCCCGTCCGCACA<br>TTTTGGTTCTGTGGCTGGGGCGCTGGAACTGACACCGCACAACGCCATCCA

	CCGGCAAGTTGGTGCGACACCGGGCGGGTTGATGGCCGATCCTGACCTGG<br>CCGCACT-3’
sequence 7	5’-CGCCTACCTGTACTACTTCGAGCAATACCTCCTGGACCAGGAGCCGACCG<br>TGGGCCTGCCCTGGTGGGACTGGTCCGCCCAGCAGGGGATCCCGCCGGCG<br>TACGCCGCGTCCACCCTCCCGGGTGGAGCAGCCAACCCTCTGGCCTCGGCA<br>CCCGTCAGAGGCATCCCGGCCAGCCAGTTCCACGCCGTGAGCGAGCAACC<br>CATCACCCGGACCCGGCGGGCACCGGGCTCGCCCGACGGGCTGCCCAGCG<br>CCGCCGACGTGGCGCAGGTGCTGGCGCTCGACGACTTCATGGACTTCACCC<br>AGGGTCTCGAGGGGCTGCACAACCAGGTCCACGTGTGGGTCGGGGCCACC<br>ATGTCCCAGATCCCGGTGGCGGCGTT-3’
sequence 8	5’-GGCCTACCTGTACTACTTCGAGCAGCACCTGCTGGACCAGATGCCGTCCG<br>GCCAGTTGGTCAGCTTGCCCTGGTGGGACTGGTCCACCCAGGCCGGTATCC<br>CGGCCGCGTACGCCGAGGCCAAGCTGCCCGACGGCGCGGCCAACCCGCTG<br>GCCGGCGCGCCCATCTCAGGCATCCCCGCGGCCCAGTTCAACGACGAGGA<br>CGTGCCGCAGGCCGAGCACACCTTCCGCCAGCCGGGGCCGACCGGCCCGA<br>ACCAGGGACCGGCCGGGCTGCCCAGCACCCGGCAAGTCGCCACGGTCCTG<br>GCCCTGAAGGACTTCGATGACTTCACCGTGCAGCTAGAGGACCTGCACAAC<br>CAGGTGCACGTCTGGGTCGGCGGGACGATGAGCGAGATCCCGCTGGCCGC<br>CTT-3’
sequence 9	5’-GATCTACCTGTTGCGGATGGAGGAGCTGCTCATGACGGTCGATCCGACCG<br>TGTGCCTTCCCTACTGGAAGTCGAGCGAGGAACAGGCGTTTCCCTCGTGGC<br>TCCTCGGCTTCACCCCGACGGTGAACCTGATCGGCGGGCCGCACACGGTCA<br>CCCGGAACATCGGCGCGTTCGCTCTGCTGCCGGATGCGGCGGCCGTGGCG<br>GCTGTCATGGCGAACGCCACCTTCAACCCGTTCGCCGGGGGGCTCGAGGG<br>AGTGCACAACTCCGGGCACGTCTGGGTCGGGGGCTCCATGATGAGCATTCC<br>CACCGCCCCCTG-3’
sequence 10	5’-GATCTACCTGTTGCGGATGGAGGAGTTGCTCATGATGGTTGACCCGACCG<br>TGTGCATCCCCTACTGGAAATCGAGCGAGGAACAATCATTCCCGTCGTGGCT<br>CGTCGGTTTCACACCGACTGTGACGCTGATGAGCGGACCTCACACGGTCAC<br>GCGAAATATCGGCATGTTCGCATTCTTACCGAACGCTGCGGCTGTGACGGCA<br>GCGCTGGCCAATGGTACCTTCAACACGTTCGCGCCGGCGCTCGAAGGGATT<br>CACAATTCCGGGCATGTCTGGGTGAGTGGTTCCATGGGAAGCATCCAGTTTG<br>CACCGTG-3’
sequence 11	5’-CGCGTACCTTGCCTGGTTCGAGGGGACGATCCGGAAGCTCGCCGGCGAT<br>GATACGTGGGGGCTTCCCTACTGGAACTACTCCGACCCCGACAACCCCGAC<br>GCCGCGTTCCTTCCGGCGGAGTTCCGGGTGCCGACCAGGACCGTCGACGG<br>CGAGGCCGTGCCGAACCCGCTGTTCGATCCGACCCGGAACGAGGGTCCCC<br>TTCCAGCCGAGGACATCGACATCGTGCCGGCGCTCACCGAACCCCGGTTCG<br>TCGGCGGGGTTCCGGATGTCGGATTCGGCGGCACCGACCGTGACCGTCGC<br>TTCGGTGACGTGGAGAGTACGCCGCACAACTGGGTTCACGTGGACATCGGC<br>GGACTCATGGAGAGCCCGGCCACCGCTGGCCA-3’
sequence 12	5’-CCGCTTCCTGCTGGAGTTCGAGCAGGCGCTGCAGTCCGTCGACGCCGAC<br>GTCGCCCTGCCGTACTGGGACTGGACGGCGGACCGCACGGCCGCGGCGTC<br>GCTGTGGGCCCCGGACTTCCTCGGCGGCACCGGCCGCAGCCGGGACGGAC<br>AGGTGACGGACGGGCCGTTCGCGTTCTCGGGAAGCCGCTGGCCGATCCAG<br>GTCCGGGTGGACGGCCGCAACTTCCTGCGCCGCTCCCTCGGTTCGGGCAC<br>CCGGCCGCTGCCCACCCGGGCGGAGGTGGACTCGGTGCTCGCCATGGCGA<br>CCTACGACATGCCGCCCTGGAACAGCGCCTCGGACGGCTTCCGCAACCACC<br>TGGAGGGCTGGCGGGGCGTCAACCTGCACAACCGCGTGCACGTGTGGGTC<br>GGCGGCCACATGGCGACCGGCGCGTCCCCGAA-3’
sequence 13	5’-CAAGTTCCTCATCGACTTCGAGGGCGAACTCCAGAAGGTCGACGCGTCGG<br>TGTCGATCCCTTACTGGGACTGGACCGTCGACAACACCGTCGGCTCCTCGC<br>TCTGGGCGCCGGACTTCCTCGGCGGCACCGGTCGCCCCCTGGACGACCAG<br>GTGATGGACGGCCCCTTCGCGTACTCCGCCGGCCAGTGGACGATCAACGTC<br>AAGGTCGACAGCCGTGATTTCCTCGCCCGCAATCTCGCCTATCGGGTTCCCA<br>CGCTCCCGCCCCGGTCCGACGTCGACGCCGCTCTCGCCCTTCCCACCTACG<br>ACTCCGCGCCGTTCCGCGAGGGCTCGGGCGGCTTCCGCTCCGCACTCGAA<br>GGCTCGGCCGGATACATGAGCATGCACAACCGGGTGCACACCTGGATCTGG<br>GGCCAGATGGAGACGAGCGTCTCCCCGAA-3’
sequence 14	5’-CGAGTACGTGCGCCGGTTCGAGCAGGATCTTCAAGCGATCGACCCCTCG<br>GTCAGCGTGCCGTACTGGGACTGGACCCAGTCCAACGTCAATGCTGCCGGC

	ACTGAGAGCCTGATCTGGCGCGACGACTTCATGGGAGGACCGGGGCAGGC<br>CGGCACCGGGCTGGTCGCGGACGGCCCGTTCGCCGGCTGGGGACTGCGC<br>CGCAGCGCCTTCAACATCTTCGGTTTCCCCGGTACGGGAGGCACCATCGCC<br>ACCCACATGGCCAATCCGAGTTACACGGGCTTCCGCGCGGTGGAGGGGCC<br>GCATGGCGCCGCCCACGTGTGGGTCGGCGGCTTCGTCGGAAACGCATCGA<br>TCGCACCGCG-3’
sequence 15	5’-GGCCTACCTGTTCCATTTTGAACAGATCTGCCGCGAGCTCTCCGGCGACG<br>ACGGGTTCGCGCTGCCGTACTGGAACTGGACCGCCAACCGGCAGATCCCG<br>GCGGTATTCTGGGACACGTCCTCGCCGTTGTACCACGCCAACCGGGTGGCC<br>GACCAGGACAGTGAGGCCGCCGAGACAGCGGTCGACCAGGGCGTTATCAA<br>CACCGCGCTGGCACCTACCAACTTCATCCTGTTCGCGGGCCAGGCGGTCGG<br>GCTCAACCATCCGCCCAACTTCGGGCCCGGCATGGGACCGTTGGAGAGCG<br>GCCCCCACAACTACATCCACAACTTCGTCGGAGGCACGATGGCAAGCTTCGT<br>CTCCCCGGG-3’
sequence 16	5’-GGCTTACCTGTTCTACTTCGAACAGATATGCCGGGAGCTTTCCGGCGACG<br>ACGACTTCGCGCTGCCGTACTGGAACTGGACCGCCGACCGGCGGGTCCCG<br>CCGGTCCTCTGGAACACCTCGTCGCCGCTGTTTCATGCCAATCGGCTAGCC<br>GAGGCGGACAGCCAGGCGGCGGAGGCGTCGGTGGATCAGGACGCTGTCGA<br>CACCGCACTGGCGCCGACCAACTTCATCTTGTTCGCTGGCGATCAGGCCGG<br>GTTGAACGCTCCACCCACCCACGGCACAGGGTTCGGCCCTTTGGAGGCCGG<br>GCCACACAATGATGTGCACGTGTTTGTGGGTGGCGACATGGGACAGGTCGC<br>GCTCGCGGCGCG-3’
sequence 17	5’-GATGTACCTCTACTGGTTCGAGCAGATCGTGCGGGACCAGTCGGGCTATG<br>CCGACTTCGCGCTCCCCTATTGGGATTACTCCAACCCTTCCCAGCGGTACTT<br>GCCGGAACCGTTTCGCGATCCCAATAATCCGCTCTATGTCAGCAAGAGGCG<br>GCCAAGCGTGAACCTGAGAGGCCCTAACGATCCGCCACCCGATGGTTCCTG<br>GTTCAACTACTGCAACGGCTTATCCCAGAGCTCGTTTGGCGATTATGATGGA<br>ACTCCTGGAGCATCCGATCGCCTCGAGGGCGATGTGCATGACAACATCCAT<br>GGCTGGGTAGGCGGCGGTACCCAGATGGATCCTGGGATCATGTCCGCTGTG<br>TCGACATCGGCCCA-3’
sequence 18	5’-GATGTACCTGTTCCACTTCGAGCAGGTTCTCCGCGGCATTCTCGCCGAGA<br>CGAACGATCCGTTGCTCGAGCCCGAGACCACGGAGTCGTGGGCCCTCCCGT<br>ACTGGAACTACCAGCTCCCGGCCCACCGCTTCCTCCCGCGGGCGTTCCGGG<br>AGACGACGCTTCCCGATGGGAGATCGAACCCGCTGGCGGACGCCCGGCGG<br>TTCGCCCACGTCCAGTCGGGCCAAGTGGGTCTCAGCGACGACCAGGTCGAC<br>TTCTCCGGATGGTGGACCGAGTCGGTGTTCACGCTGCCGACGGCGCCGTCG<br>TTCGGCGGATCGGACACCGCTGGACCCCGCCACCGACCGGTCGACTTCGA<br>GAGCGCGGGCGCGCTCGAGGTGACGCCGCACGGCGATGTCCACATGTACG<br>TCGGACCGGACATGCGGTTGTTCACGACGGCCGGGCT-3’
sequence 19	5’-CAGATTCCTCATACAGTTCGAGCAGGCGCTCCAGGAGATCGACCCCGCCG<br>TCGCCCTGCCCTACTGGGACTGGACGGTGGACCGCACCGTCGCGTCCTCCC<br>TGTGGGCACCGGACTTCCTCGGCGGCACCGGCCGTGCCAGGGACGGGCAG<br>GTCGCCGAGGGGCCGTTCGCCGTCAGCAGCGGCAGATGGCCGGTCACGGT<br>GACGGTCGACGGGCGCGGCTTCCTCCGCCGGGCCCTCGGCTCCGGCGTAG<br>CGCAACTGCCGACCCGGGCGGAGGTCGAATCGGTCCTCGCCATGCCCACG<br>TACGACACCGCGCCCTGGAACAGCGCGTCGGACGGCTTCCGCAACCACCTC<br>GAAGGCTGGCGCGGCGTCAATCTCCACAACCGGGTGCACGTGTGGGTGGG<br>CGGCCAGATGGCCACCGGCGCCTCGCCCAA-3’

Table S2. Partial TYR nucleotide sequences. Nucleotide sequences identified from peat samples covering the sequence between the two degenerated type III copper protein primers (Table S1).

	BLAST match	identity (%)	host organism
sequence 9	A0A0C3RMP7	78.8	*Nitrosospira sp. NpAV*
sequence 10	A0A0C3RMP7	82.7	*Nitrosospira sp. NpAV*
sequence 11	A0A2T8F577	90.6	*Nocardioides gansuensis*
sequence 12	A0A1Z1WBL2	87.4	*Streptomyces alboflavus*
sequence 13	G0PQZ3	91.0	*Streptomyces sp. ACT-1*
sequence 14	A0A4P9J8U1	77.3	*Citricoccus sp. SGAir0253*
sequence 19	A0A2S3Y8X7	100.0	*Streptomyces sp. ZL-24*

Table S3. High identity matches (> 75 %) identified by a BLAST search. The UniProt identifiers for the matches, their identity level, and the respective host organisms are listed.

sequence 1	AYLYYFERILRWAANDPTLALPYWNYHDENQRTIPQAYRDATFGQDKAPNPLYLSANARFTDKDGKPQLFPMRDADLNQGLTQLAAPYVSTDALLATAFTASPPAPVNTTFGSAWACDQTCACAGGALERIP**HN**AV**HN**AIGGALVTTGGSFWVGFMGDITTSA
sequence 2	MYVLNFERIVARHVESLGGPADWALPYWNYTTTNPVTLALPPAFRSPLLPTGGPNPLYVALRNPVANAGSPVLGRRDVSLNCLSTSGTTRPGDFFGGSPPDHNGSLPGALELTP**HN**AI**HN**QVGKTPGGWMQDPDLAA
sequence 3	MFLCYFELLIRQASGRPDFTLPYWDYTNAKHPESAALPDEFHWPGVVSNSLYRVNRNRKANAGKPIDTIGPTGLNLGSLSERTYRDHGAAMGFGQNLDVYL**HS**DV**HI**LTGNSLGMGSIPWAA
sequence 4	GYLIAFEKVIRAAVVAAGGPADWALPYWNYFKPNQNQLPPAFATPDWPDGAGDNPLYIKQRHGPRNNGNVYVLLSKVNLKALGDNNFIGAGTGGSLGFGGVVTGFSHQHSPHGGIESQP**HD**QV**HG**LVGGADPTTGLPGLMSDPDTAG
sequence 5	MYVLNFERIVAMHVASLGGPADWALPYWNYTTTNPATLELPPAFRDPVLATGAPNHLYVALRNPLANAGGAVLGPRDVALNCLSASGTTIPGGFFGGAPPDHSGHLGGALELTP**HN**AI**HN**QVGKTLGGLMQDPDLAA
sequence 6	MYILNFERIVARHVVSLGGPADWALPYWNYTTSDTATLALPPAFRNPALPGGAPNPLHVALRNPVANAGGSVLGPHDVDLTCLNASGTTIPGGFFGGAPSAHFGSVAGALELTP**HN**AI**HR**QVGATPGGLMADPDLAA
sequence 7	AYLYYFEQYLLDQEPTVGLPWWDWSAQQGIPPAYAASTLPGGAANPLASAPVRGIPASQFHAVSEQPITRTRRAPGSPDGLPSAADVAQVLALDDFMDFTQGLEGL**HN**QV**HV**WVGATMSQIPVAA
sequence 8	AYLYYFEQHLLDQMPSGQLVSLPWWDWSTQAGIPAAYAEAKLPDGAANPLAGAPISGIPAAQFNDEDVPQAEHTFRQPGPTGPNQGPAGLPSTRQVATVLALKDFDDFTVQLEDL**HN**QV**HV**WVGGTMSEIPLAA
sequence 9	IYLLRMEELLMTVDPTVCLPYWKSSEEQAFPSWLLGFTPTVNLIGGPHTVTRNIGAFALLPDAAAVAAVMANATFNPFAGGLEGV**HN**SG**HV**WVGGSMMSIPTAP
sequence 10	IYLLRMEELLMMVDPTVCIPYWKSSEEQSFPSWLVGFTPTVTLMSGPHTVTRNIGMFAFLPNAAAVTAALANGTFNTFAPALEGI**HN**SG**HV**WVSGSMGSIQFAP
sequence 11	AYLAWFEGTIRKLAGDDTWGLPYWNYSDPDNPDAAFLPAEFRVPTRTVDGEAVPNPLFDPTRNEGPLPAEDIDIVPALTEPRFVGGVPDVGFGGTDRDRRFGDVESTP**HN**WV**HV**DIGGLMESPATAG
sequence 12	RFLLEFEQALQSVDADVALPYWDWTADRTAAASLWAPDFLGGTGRSRDGQVTDGPFAFSGSRWPIQVRVDGRNFLRRSLGSGTRPLPTRAEVDSVLAMATYDMPPWNSASDGFRNHLEGWRGVNL**HN**RV**HV**WVGGHMATGASP
sequence 13	KFLIDFEGELQKVDASVSIPYWDWTVDNTVGSSLWAPDFLGGTGRPLDDQVMDGPFAYSAGQWTINVKVDSRDFLARNLAYRVPTLPPRSDVDAALALPTYDSAPFREGSGGFRSALEGSAGYMSM**HN**RV**HT**WIWGQMETSVSP

sequence 14	EYVRRFEQDLQAIDPSVSVPYWDWTQSNVNAAGTESLIWRDDFMGGPG<br>QAGTGLVADGPFAGWGLRRSAFNIFGFPGTGGTIATHMANPSYTGFRA<br>VEGP**HG**AA**HV**WVGGFVGNASIAP
sequence 15	AYLFHFEQICRELSGDDGFALPYWNWTANRQIPAVFWDTSSPLYHANRV<br>ADQDSEAAETAVDQGVINTALAPTNFILFAGQAVGLNHPPNFGPGMGPL<br>ESGP**HN**YI**HN**FVGGTMASFVSP
sequence 16	AYLFYFEQICRELSGDDDFALPYWNWTADRRVPPVLWNTSSPLFHANRL<br>AEADSQAAEASVDQDAVDTALAPTNFILFAGDQAGLNAPPTHGTGFGPL<br>EAGP**HN**DV**HV**FVGGDMGQVALAA
sequence 17	MYLYWFEQIVRDQSGYADFALPYWDYSNPSQRYLPEPFRDPNNPLYVS<br>KRRPSVNLRGPNDPPPDGSWFNYCNGLSQSSFGDYDGTPGASDRLEG<br>DV**HD**NI**HG**WVGGGTQMDPGIMSAVSTSA
sequence 18	MYLFHFEQVLRGILAETNDPLLEPETTESWALPYWNYQLPAHRFLPRAF<br>RETTLPDGRSNPLADARRFAHVQSGQVGLSDDQVDFSGWWTESVFTLP<br>TAPSFGGSDTAGPRHRPVDFESAGALEVTP**HG**DV**HM**YVGPDMRLFTTA<br>G
sequence 19	RFLIQFEQALQEIDPAVALPYWDWTVDRTVASSLWAPDFLGGTGRARDG<br>QVAEGPFAVSSGRWPVTVTVDGRGFLRRALGSGVAQLPTRAEVESVLA<br>MPTYDTAPWNSASDGFRNHLEGWRGVNL**HN**RV**HV**WVGGQMATGASP

Table S4. Partial TYR amino acid sequences. Amino acid sequences identified from peat samples covering the sequence between the two degenerated primers (Table S1). His_{B1} and His_{B2} are highlighted in brown, the 1st activity controller (His_{B1}+1) is highlighted in cyan and the 2nd activity controller (His_{B2}+1) is highlighted in magenta.

	2	3	4	5	6	7	8	9	10	11	12	13	14	15	16	17	18	19
1	29.0	18.4	26.4	28.4	26.6	21.4	21.5	18.3	16.5	25.0	20.7	21.1	19.8	24.5	21.5	25.6	22.8	19.2
2		26.3	37.6	83.2	77.4	20.7	24.0	13.5	12.6	28.1	20.4	19.6	23.1	25.9	29.5	31.9	29.0	20.3
3			21.5	29.3	27.9	25.6	26.5	19.4	22.0	23.6	21.3	14.9	17.0	22.0	26.1	30.0	19.6	24.2
4				37.1	36.4	19.6	19.4	19.6	14.8	31.4	17.8	18.9	22.2	25.8	23.8	25.2	25.6	16.6
5					78.8	20.7	24.7	16.6	18.3	27.4	23.1	20.1	22.1	26.4	25.7	33.1	29.0	20.8
6						22.5	26.0	15.8	12.2	30.8	22.5	18.1	19.6	24.8	27.0	29.5	30.3	17.9
7							62.7	34.4	31.2	18.7	26.9	21.8	22.8	25.2	30.2	22.1	24.0	28.4
8								28.2	25.2	20.8	25.0	20.3	23.6	22.1	28.7	20.0	22.4	23.8
9									78.9	19.7	25.0	20.0	25.0	21.1	23.6	19.1	12.7	25.7
10										17.8	23.6	22.8	25.0	17.9	20.3	17.3	14.2	25.0
11											21.7	16.6	17.5	28.7	28.7	27.4	30.7	19.2
12												55.6	29.5	25.3	26.2	19.6	18.0	80.4
13													27.3	19.9	19.3	18.0	16.4	58.3
14														21.4	24.6	18.8	19.3	32.4
15															67.8	26.3	27.3	24.7
16																23.3	29.5	26.7
17																	24.8	20.1
18																		18.3

Table S5. Identity matrix of the 19 identified TYR partial amino acid sequences. Identity values (%) were calculated using the Clustal Omega program implemented in the UniProt alignment tool (https://www.uniprot.org/align/).

full-length sequence of *Sz*TYR	5’-ATGACCGTACGCAAGAACCAGGCGAGCCTGACCGCCGCC GAGAAGCGCACCTTCGTCGACGCCGTCCTGGAGCTCAAGCG CACCGGCCGCTACGACTCGTTCGTCACCACGCACAACGCCT TCATCATGAGCGACACCGACAACGGTGACCGGGTGGGTCAC CGGTCGCCCTCCTTCCTGCCCTGGCACCGCAGATTCCTCATA CAGTTCGAGCAGGCGCTCCAGGAGATCGACCCCGCCGTCGC CCTGCCCTACTGGGACTGGACGGTGGACCGCACCGTCGCGT CCTCCCTGTGGGCACCGGACTTCCTCGGCGGCACCGGCCGT GCCAGGGACGGGCAGGTCGCCGAGGGGCCGTTCGCCGTCA GCAGCGGCAGATGGCCGGTCACGGTGACGGTCGACGGGCG CGGCTTCCTCCGCCGGGCCCTCGGCTCCGGCGTAGCGCAAC TGCCGACCCGGGCGGAGGTCGAATCGGTCCTCGCCATGCCC ACGTACGACACCGCGCCCTGGAACAGCGCGTCGGACGGCTT CCGCAACCACCTCGAAGGCTGGCGCGGCGTCAATCTCCACA ACCGGGTGCACGTGTGGGTGGGCGGCCAGATGGCCACCGG CGCCTCGCCCAACGACCCGGTCTTCTGGCTGCACCACGCGT TCATCGACCGGCTCTGGGCCCAGTGGCAGGCACGCCACCCC CGCTCCACCTACCTGCCGGCCGCGAGGACCAGGAACGTCGT GGGCCTGGATGACGTCATGCGCCCCTGGAACGACGTGACGC CCGCCGACATGCTGGATCACACCCGGCACTACACGTACGAC ACGATGGCGTAG-3’
full-length sequence of the caddie protein (A0A2S3Y8X5)	5’-ATGTCTCGAATCACCCGTCGCCATGCCCTGGGGGCCGCTG CCGCGACCGCTCTGACCGGCCTCGCCCTGGCCGGCCTCGC GCGGACGGCCGGAGCCACGTCCGCCCCCCGGGCCACCGGC CACCAGCCCCCCGAGGGGCACGCCGGGCACGACGGCCCGC AGCCCTTCAGCGAGACCTTCCAGGGGCGCCGCATCGAGGGC GCCCCGTCGCACGCCGACGGGCACCACGGCGGCTACGCC GTACGGATCGACGGCGAGGAACTGCACGTGATGCGGAACGC CGACAGCACCTGGGTCAGCGTCATCAACCACTACGAGACGTT CACGACCCCGCGCGCGGTCGCCCGGGCCGCCGTGATCGAG CTCCAGGGCGCCCGACTCGTACCCCTGGCCTGA-3’
full-length sequence of the codon-optimized (towards the codon usage of *E. coli*) caddie protein	5’-ATGTCGCGCATTACGCGTCGTCATGCCCTGGGTGCAGCCG CAGCAACCGCTCTGACGGGTTTGGCGCTTGCGGGCTTAGCG CGTACTGCGGGTGCGACTTCAGCCCCACGTGCGACAGGCCA TCAGCCTCCGGAAGGCCATGCTGGGCATGATGGACCGCAGC CGTTTAGCGAAACCTTTCAGGGTCGCCGCATTGAAGGCGCTC CCAGTCACGCGGATGGCCACCATGGAGGCTATGCGGTTCGC ATCGATGGGGAGGAACTGCACGTGATGCGCAATGCCGACTC TACGTGGGTGTCCGTCATCAACCACTACGAAACCTTCACCAC CCCACGGGCAGTTGCGCGCGCTGCCGTGATTGAGCTGCAAG GTGCACGTCTGGTACCGCTCGCCTAA-3’

Table S6. Full-length sequences of MelC2 (*Sz*TYR), MelC1 (caddie protein) and MelC1 codon-optimized (caddie protein).

**16S RNA identified in this study**
5’-AGTCGAACGATGAAGCCGCTTCGGTGGTGGATTAGTGGCGAACGGGTGAGTAACACG<br>TGGGCAATCTGCCCTTCACTCTGGGACAAGCCCTGGAAACGGGGTCTAATACCGGATAA<br>TACTCTGTTCCGCATGGAACGGGGTTGAAAGCTCCGGCGGTGAAGGATGAGCCCGCGG<br>CCTATCAGCTTGTTGGTGGGGTAATGGCCTACCAAGGCGACGACGGGTAGCCGGCCTG<br>AGAGGGCGACCGGCCACACTGGGACTGAGACACGGCCCAGACTCCTACGGGAGGCAG<br>CAGTGGGGAATATTGCACAATGGGCGAAAGCCTGATGCAGCGACGCCGCGTGAGGGAT<br>GACGGCCTTCGGGTTGTAAACCTCTTTCAGCAGGGAAGAAGCGCAAGTGACGGTACCT<br>GCAGAAGAAGCGCCGGCTAACTACGTGCCAGCAGCCGCGGTAATACGTAGGGCGCAAG<br>CGTTGTCCGGAATTATTGGGCGTAAAGAGCTCGTAGGCGGCTTGTCACGTCGGATGTGA<br>AAGCCCGGGGCTTAACCCCGGGTCTGCATTCGATACGGGCTAGCTAGAGTGTGGTAGG<br>GGGAGATCGGAATTCCTGGTGTAGCGGTGAAATGCGCAGATATCAGGAGGAACACCGG<br>TGGCGAAGGCGGATCTCTGGGCCATTACTGACGCTGAGGAGCGAAAGCGTGGGGAGC<br>GAACAGGATTAGATACCCTGGTAGTCCACGCCGTAAACGTTGGGAACTAGGTGTTGGCG<br>ACATTCCACGTCGTCGGTGCCGCAGCTAACGCATTAAGTTCCCCGCCTGGGGAGTACG<br>GCCGCAAGGCTAAAACTCAAAGGAATTGACGGGGGCCCGCMCAAGCAGCGGAGCATGT<br>GGCTTAATTCGACGCAACGCGAAGAACCTTACCAAGGCTTGACATWTACCGGAAAGCAT<br>CMGAGATGGTGCCCCCCTTGTGGTCGGTATACAGGTGGTGCATGGCTGTCGTCAGCTC<br>GTGTCGTGAGATGTTGGGTTAAGTCCCGCAACGAGCGCAACCCTTGTCCTGTGTTGCCA<br>GCATGCCCTTCGGGGTGATGGGGACTCACAGGAGACCGCCGGGGTCAACTCGGAGGA<br>AGGTGGGGACGACGTCAAGTCATCATGCCCCTTATGTCTTGGGCTGCACACGTGCTACA<br>ATGGCCGGTACAATGAGCTGCGATACCGTGAGGTGGAGCGAATCTCAAAAAGCCGGTC<br>TCAGTTCGGATTGGGGTCTGCAACTCGACCCCATGAAGTCGGAGTCGCTAGTAATCGCA<br>GATCAGCATTGCTGCGGTGAATACGTTCCCGGGCCTTGTACACACCGCCCGTCACGTCA<br>CGAAAGTCGGTAACACCCGAAGCCGGTGGCCCAACCCGTAAGGGAGGGAG-3’
**16S RNA from *Streptomyces* sp. ZL-24 (GenBank Accession number: MH700447.1)**
5’-AGTCGAACGATGAAGCCGCTTCGGTGGTGGATTAGTGGCGAACGGGTGAGTACACGT<br>GGGCAATCTGCCCTTCACTCTGGGACAAGCCCTGGAAACGGGGTCTAATACCGGATAAT<br>ACTCTGTTCCGCATGGAACGGGGTTGAAAGCTCCGGCGGTGAAGGATGAGCCCGCGGC<br>CTATCAGCTTGTTGGTGGGGTAATGGCCTACCAAGGCGACGACGGGTAGCCGGCCTGA<br>GAGGGCGACCGGCCACACTGGGACTGAGACACGGCCCAGACTCCTACGGGAGGCAGC<br>AGTGGGGAATATTGCACAATGGGCGAAAGCCTGATGCAGCGACGCCGCGTGAGGGATG<br>ACGGCCTTCGGGTTGTAAACCTCTTTCAGCAGGGAAGAAGCGCAAGTGACGGTACCTGC<br>AGAAGAAGCGCCGGCTAACTACGTGCCAGCAGCCGCGGTAATACGTAGGGCGCAAGCG<br>TTGTCCGGAATTATTGGGCGTAAAGAGCTCGTAGGCGGCTTGTCACGTCGGATGTGAAA<br>GCCCGGGGCTTAACCCCGGGTCTGCATTCGATACGGGCTAGCTAGAGTGTGGTAGGGG<br>AGATCGGAATTCCTGGTGTAGCGGTGAAATGCGCAGATATCAGGAGGAACACCGGTGG<br>CGAAGGCGGATCTCTGGGCCATTACTGACGCTGAGGAGCGAAAGCGTGGGGAGCGAAC<br>AGGATTAGATACCCTGGTAGTCCACGCCGTAAACGTTGGGAACTAGGTGTTGGCGACAT<br>TCCACGTCGTCGGTGCCGCAGCTAACGCATTAAGTTCCCCGCCTGGGGAGTACGGCCG<br>CAAGGCTAAAACTCAAAGGAATTGACGGGGGCCCGCACAAGCAGCGGAGCATGTGGCT<br>TAATTCGACGCAACGCGAAGAACCTTACCAAGGCTTGACATATACCGGAAAGCATCAGA<br>GATGGTGCCCCCCTTGTGGTCGGTATACAGGTGGTGCATGGCTGTCGTCAGCTCGTGT<br>CGTGAGATGTTGGGTTAAGTCCCGCAACGAGCGCAACCCTTGTTCTGTGTTGCCAGCAT<br>GCCTTTCGGGGTGATGGGGACTCACAGGAGACTGCCGGGGTCAACTCGGAGGAAGGT<br>GGGGACGACGTCAAGTCATCATGCCCCTTATGTCTTGGGCTGCACACGTGCTACAATGG<br>CCGGTACAATGAGCTGCGATGCCGTGAGGCGGAGCGAATCTCAAAAAGCCGGTCTCAG<br>TTCGGATTGGGGTCTGCAACTCGACCCCATGAAGTCGGAGTTGCTAGTAATCGCAGATC<br>AGCATTGCTGCGGTGAATACGTTCCCGGGCCTTGTACACACCGCCCGTCACGTCACGAA<br>AGTCGGTAACACCCGAAGCCGGTGGCCCAACCCCTTGTGGGAGGGAG-3’

Table S7. 16S RNA sequence. Detailed information about the experimental setup is provided in the supporting Materials and methods section.

**mass calculated (Da)**	**mass measured (Da)**	**Δ (Da)**
30892.3	30891.8 ± 0.64	-0.5

Table S8. Calculated and measured molecular mass of recombinantly expressed *Sz*TYR. Detailed information about the experimental setup is provided in the Materials and methods section.

**pH value**	**buffer**	**T_m value**
**7**	TRIS-HCl	66.9 ± 1.0
**8**	TRIS-HCl	68.2 ± 1.4
**9**	TRIS-HCl	67.3 ± 1.3
**9**	CAPS	67.3 ± 1.3
**10**	CAPS	66.3 ± 0.9
**11**	CAPS	65.6 ± 1.1

Table S9. Melting temperatures (T_m values) of *Sz*TYR. T_m values were determined at different pH values by non-linear curve fitting of the absorption values determined *via* a thermofluor assay (Figure S10) to the Boltzmann equation (Huynh 2015, 11 in the supporting information). Measurements were performed in triplicates. T_m values are reported ± one standard deviation. *Sz*TYR was incubated in 50 mM Tris(hydroxymethyl)aminomethane (TRIS; pH 7.0, 8.0, 9.0) or 50 mM 3-(Cyclohexylamino)-1-propanesulfonic acid (CAPS; pH 9.0, 10.0, 11.0).

**substrate**	**µg *Sz*TYR**	**λ (nm)**	**ε_λ (M^{-1} cm^{-1})**	**reference**
tyramine	1.23	480	3300	[16]
tyrosine	1.06	475	3600	[16]
dopamine	0.0123	480	3300	[16]
L-DOPA	0.0117	475	3600	[16]
p-coumaric acid	1.0	495	2062	[16]
caffeic acid	0.008	495	2062	[16]
protocatechuic acid	1.0	454	213	[17]
gallic acid	3.2	346	4140	[17]

Table S10. Amounts of *Sz*TYR (µg), wavelengths (λ), and molar extinction coefficients (ε) used for kinetic measurements. The molar extinction coefficients at the respective wavelength have been reported previously (see references). Detailed information about the experimental setup is provided in the Materials and methods section of the main manuscript.

4. Figures

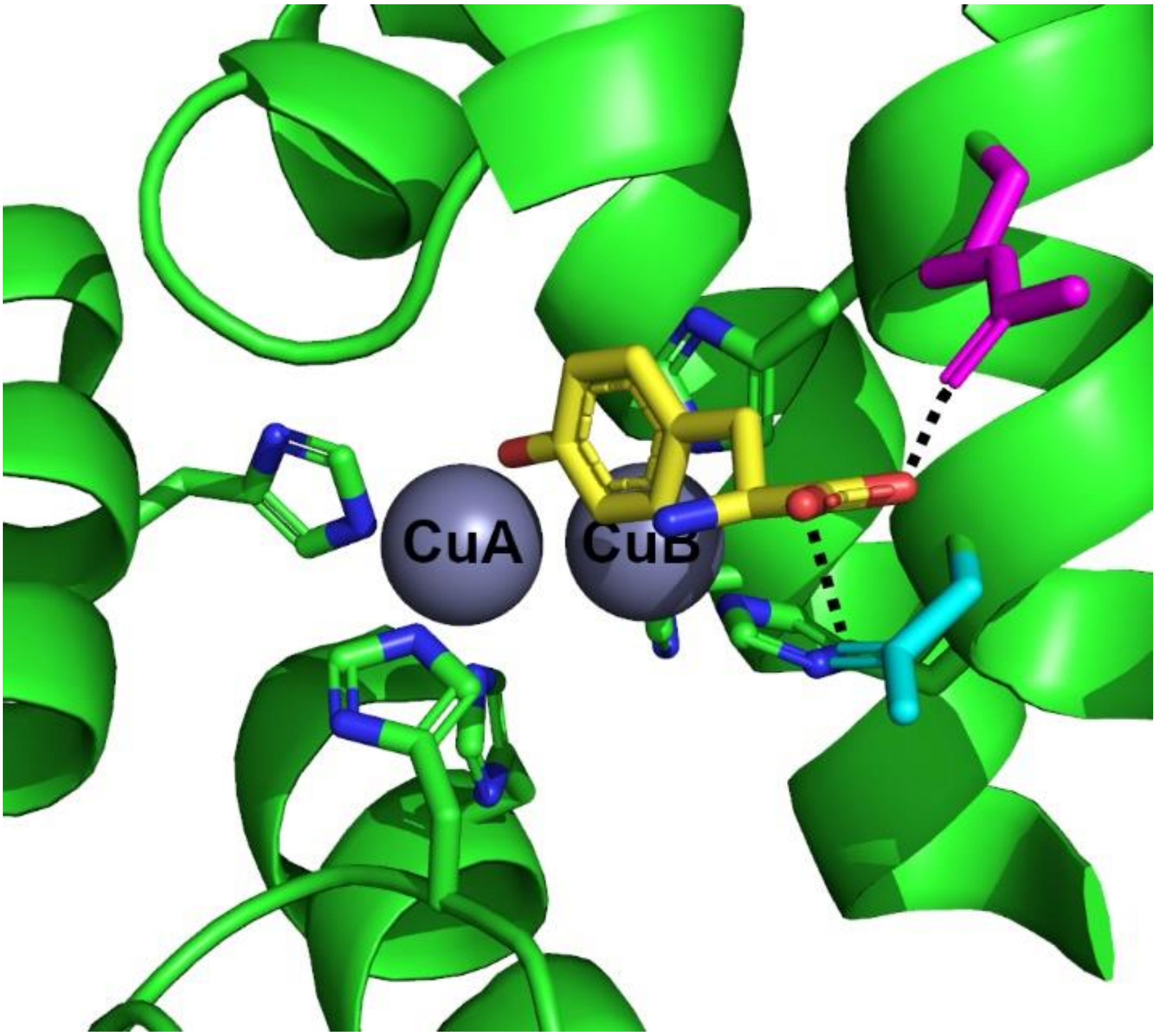

Figure S1. Active center of a bacterial TYR with tyrosine in the active site. The hydroxyl oxygen of the phenol group is oriented towards CuA and CuB. The 1st activity controller (His_{B1}+1, Asn, cyan) and the 2nd activity controller (His_{B2}+1, Arg, magenta) interact with the tail of the substrate (black dashed lines). *Bm*TYR with tyrosine crystallized in the active site featuring Zn in the position of CuA and CuB was used as a template (4P6R) [18]. The images were created using PyMOL 2.3 (https://pymol.org/2) and edited using GIMP 2.10.18 (https://www.gimp.org).

MelC 1 ATGTCTCGAATCACCCGTCGCCATGCCCTGGGGGCCGCTGCCGCGACCGCTCTGACCGGC

MelC 61 CTCGCCCTGGCCGGCCTCGCGCGGACGGCCGGAGCCACGTCCGCCCCCCGGGCCACCGGC

MelC 121 CACCAGCCCCCCGAGGGGCACGCCGGGCACGACGGCCCGCAGCCCTTCAGCGAGACCTTC

MelC 181 CAGGGGCGCCGCATCGAGGGCGCCCCGTCGCACGCCGACGGGCACCACGGCGGCTACGCC

MelC 241 GTACGGATCGACGGCGAGGAACTGCACGTGATGCGGAACGCCGACAGCACCTGGGTCAGC

MelC 301 GTCATCAACCACTACGAGACGTTCACGACCCCGCGCGCGGTCGCCCGGGCCGCCGTGATC

MelC 361 GAGCTCCAGGGCGCCCGACTCGTACCCCTGGCCTGATCCCGTCCCCGCGATTCCGCAGAA

MelC 421 CTTCTCAACCCTTTCCTGAACCGGGGAGCACCCTCATGACCGTACGCAAGAACCAGGCGA

MelC 481 GCCTGACCGCCGCCGAGAAGCGCACCTTCGTCGACGCCGTCCTGGAGCTCAAGCGCACCG

MelC 541 GCCGCTACGACTCGTTCGTCACCACG**CAC**AACGCCTTCATCATGAGCGATACCGACAACG

MelC 601 GTGACCGGGTGGGT**CAC**CGGTCGCCCTCCTTCCTGCCCTGG**CAC**CGCAGATTCCTCATAC

MelC 661 AGTTCGAGCAGGCGCTCCAGGAGATCGACCCCGCCGTCGCCCTGCCCTACTGGGACTGGA

MelC 721 CGGTGGACCGCACCGTCGCGTCCTCCCTGTGGGCACCGGACTTCCTCGGCGGCACCGGCC

MelC 781 GTGCCAGGGACGGGCAGGTCGCCGAGGGGCCGTTCGCCGTCAGCAGCGGCAGATGGCCGG

MelC 841 TCACGGTGACGGTCGACGGGCGCGGCTTCCTCCGCCGGGCCCTCGGCTCCGGCGTAGCGC

MelC 901 AACTGCCGACCCGGGCGGAGGTCGAATCGGTCCTCGCCATGCCCACGTACGACACCGCGC

MelC 961 CCTGGAACAGCGCGTCGGACGGCTTCCGCAACCACCTCGAAGGCTGGCGCGGCGTCAATC

MelC 1021 TC**CAC**AACCGGGTG**CAC**GTGTGGGTGGGCGGCCAGATGGCCACCGGCGCCTCGCCCAACG

MelC 1081 ACCCGGTCTTCTGGCTGCAC**CAC**GCGTTCATCGACCGGCTCTGGGCCCAGTGGCAGGCAC

MelC 1141 GCCACCCCCGCTCCACCTACCTGCCGGCCGCGAGGACCAGGAACGTCGTGGGCCTGGATG

MelC 1201 ACGTCATGCGCCCCTGGAACGACGTGACGCCCGCCGACATGCTGGATCACACCCGGCACT

MelC 1261 ACACGTACGACACGATGGCGTAG

Figure S2. Localization of MelC1 (caddie protein, blue) and MelC2 (tyrosinase, green) within the MelC operon from *Streptomyces sp.*. The nucleotide sequence of the caddie protein (highlighted in blue) is followed by the nucleotide sequence of the TYR (highlighted in green) with a short non-coding region spaced between the caddie protein and the TYR. The six copper coordinating histidine residues, all of which are coded by a CAC codon, are indicated in bold red letters. The conserved primer binding regions are highlighted in brown. The caddie protein and the TYR both start with ATG (start codon) and end with TAG (stop codon). The sequence has been identified within the scope of this work.

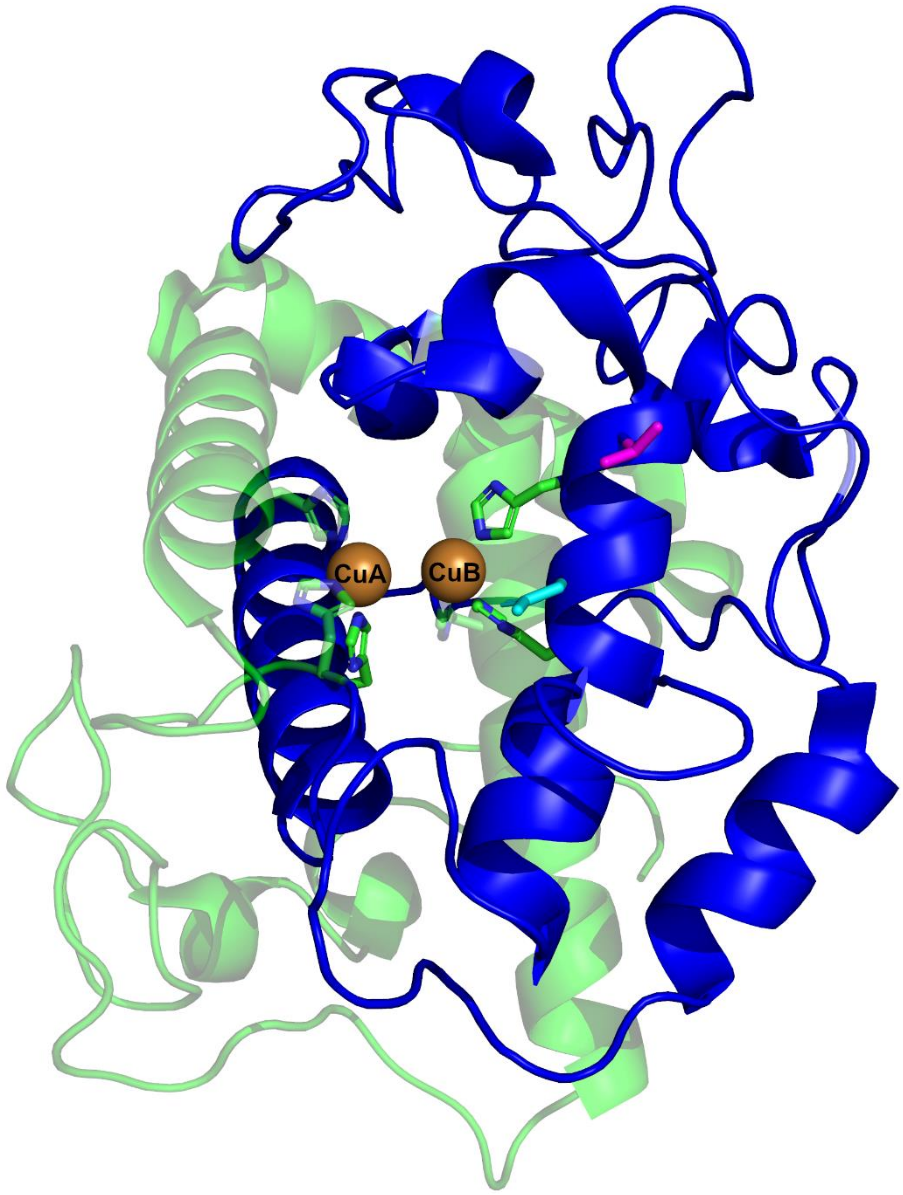

Figure S3. Sequence covered by the identified TYR partial sequences. The blue part of the sequence represents the identified partial sequence including His$_{A3}$, His$_{B1}$, His$_{B2}$, the 1st activity controller (His$_{B1}$+1, cyan sticks), and the 2nd activity controller (His$_{B2}$+1, magenta sticks). The green part represents the regions of the TYR sequence outside the primer binding sites (transparency set to 0.3). His$_{A1}$, His$_{A2}$, His$_{B3}$ are represented as sticks (transparency 0.3). The copper ions (CuA and CuB) are displayed as brown spheres. The crystal structure of the active domain of the TYR from *Streptomyces castaneoglobisporus* (*Sc*TYR, PDB entry 2zmz) was used as a model. The Figure has been created using PyMOL 2.3 (https://pymol.org/2) and edited using GIMP 2.10.18 (https://www.gimp.org).

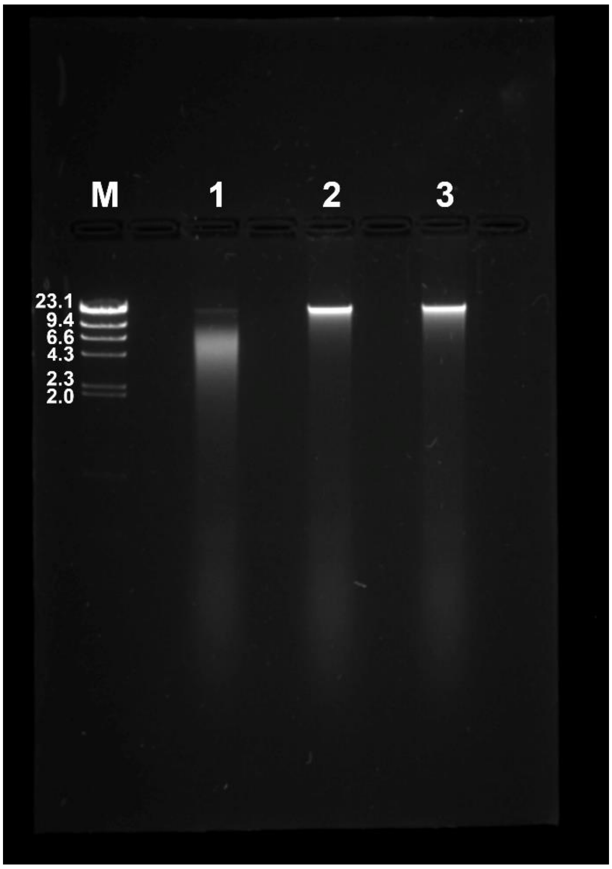

Figure S4. Metagenomic DNA extracted from soil samples. M represents the marker lane (λ DNA digested with HindIII, ThermoFisher). Molecular weights are indicated in kilobase pairs. Lane 1 represents a DNA extract prepared by cell lysis using UltraTurrax, 2 represents a metagenomic DNA extract prepared by cell lysis using freeze-thaw cycles, 3 represents a metagenomic DNA extract prepared by cell lysis using glass beads. The band at around 20 kbp was identified as DNA by a successful restriction enzyme digest (HindIII, NEB, Ipswich, USA). The Figure has been edited using GIMP 2.10.18 (https://www.gimp.org).

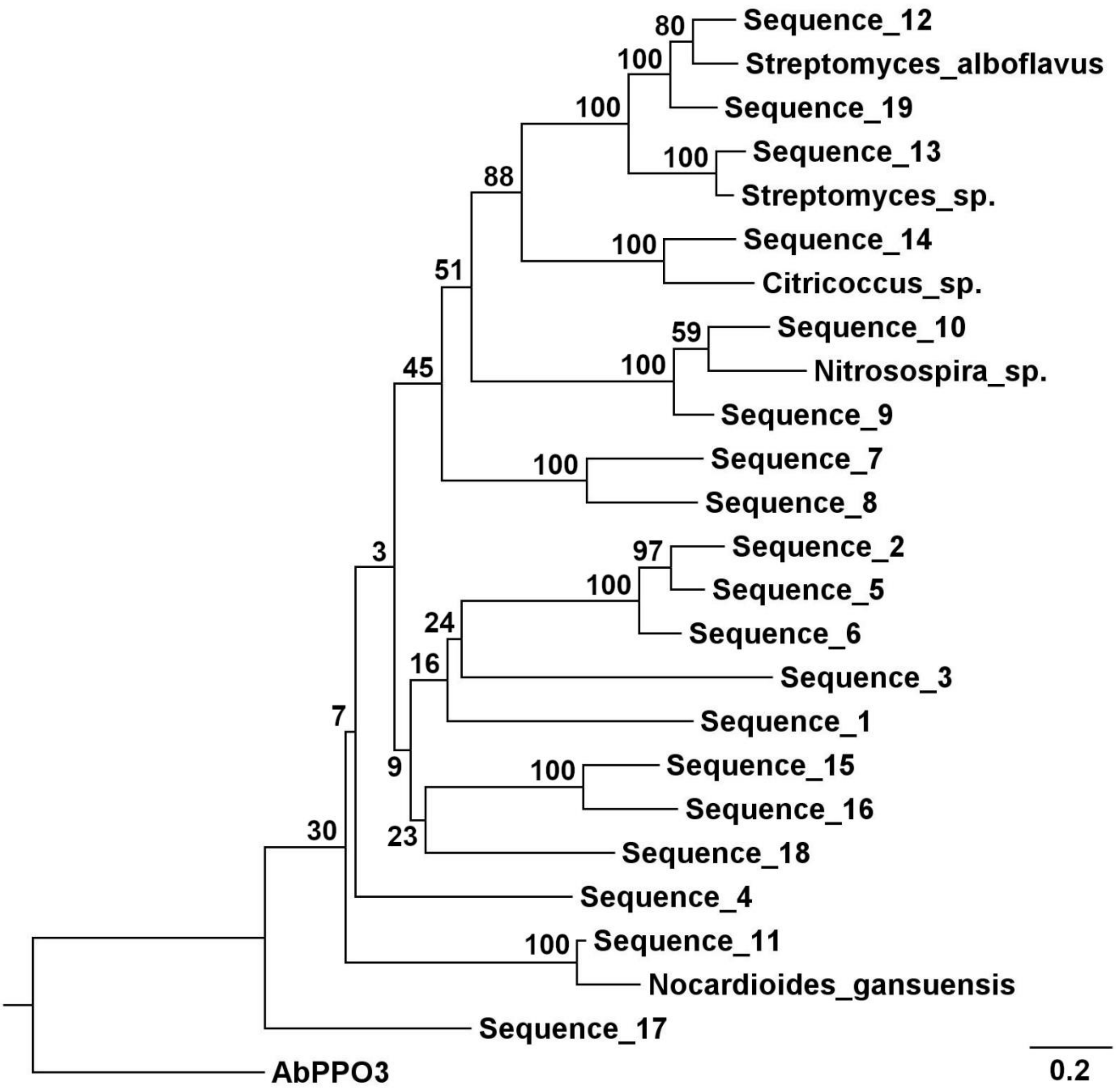

Figure S5. Phylogenetic tree of the 19 identified nucleotide sequences (Table S2). The tree was rooted to the characterized TYR sequence from *Agaricus bisporus* (*Ab*PPO3, UniProt Identifier: C7FF04) which represents a valuable outgroup due to its phylogenetic localization in a different kingdom (fungi). Sequences of TYR enzymes identified by the BLAST search (identity level > 75 %) and their respective host organisms were included (Table S3) to offer additional information about the localization of the identified partial TYR sequences in the phylogenetic spectrum (Table S2, S3). The reliability of the internal branches was assessed by bootstrapping (1000 replicates) and probability values are reported in %. Detailed information about the computational setup is provided in the Materials and methods section of the supporting information.

**Sequence 1**	LERIP**HN**AV**HN**AIGGA
**Sequence 2**	LELTP**HN**AI**HN**QVGKT
**Sequence 3**	LDVYL**HS**DV**HI**LTGNS
**Sequence 4**	IESQP**HD**QV**HG**LVGGA
**Sequence 5**	LELTP**HN**AI**HN**QVGKT
**Sequence 6**	LELTP**HN**AI**HR**QVGAT
**Sequence 7**	-LEGL**HN**QV**HV**WVGAT
**Sequence 8**	-LEDL**HN**QV**HV**WVGGT
**Sequence 9**	-LEGV**HN**SG**HV**WVGGS
**Sequence 10**	-LEGI**HN**SG**HV**WVSGS
**Sequence 11**	VESTP**HN**WV**HV**DIGGL
**Sequence 12**	--VNL**HN**RV**HV**WVGGH
**Sequence 13**	-YMSM**HN**RV**HT**WIWGQ
**Sequence 14**	-VEGP**HG**AA**HV**WVGGF
**Sequence 15**	LESGP**HN**YI**HN**FVGGT
**Sequence 16**	LEAGP**HN**DV**HV**FVGGD
**Sequence 17**	LEGDV**HD**NI**HG**WVGGG
**Sequence 18**	LEVTP**HG**DV**HM**YVGPD
**Sequence 19**	-GVNL**HN**RV**HV**WVGGQ

Figure S6. Multiple sequence alignment of a segment of the 19 partial TYR sequences (Table S4) around the activity controller residues. The 1st activity controller (His$_{B1}$+1) is highlighted in cyan, the 2nd activity controller (His$_{B2}$+1) is highlighted in magenta, His$_{B1}$ and His$_{B2}$ are highlighted in brown. The alignment shows the low level of conservation, except for the two conserved copper coordinating histidines (His$_{B1}$ and His$_{B2}$), as well as the different amino acids featured in the positions of the 1st (His$_{B1}$+1) and 2nd (His$_{B2}$+1) activity controllers.

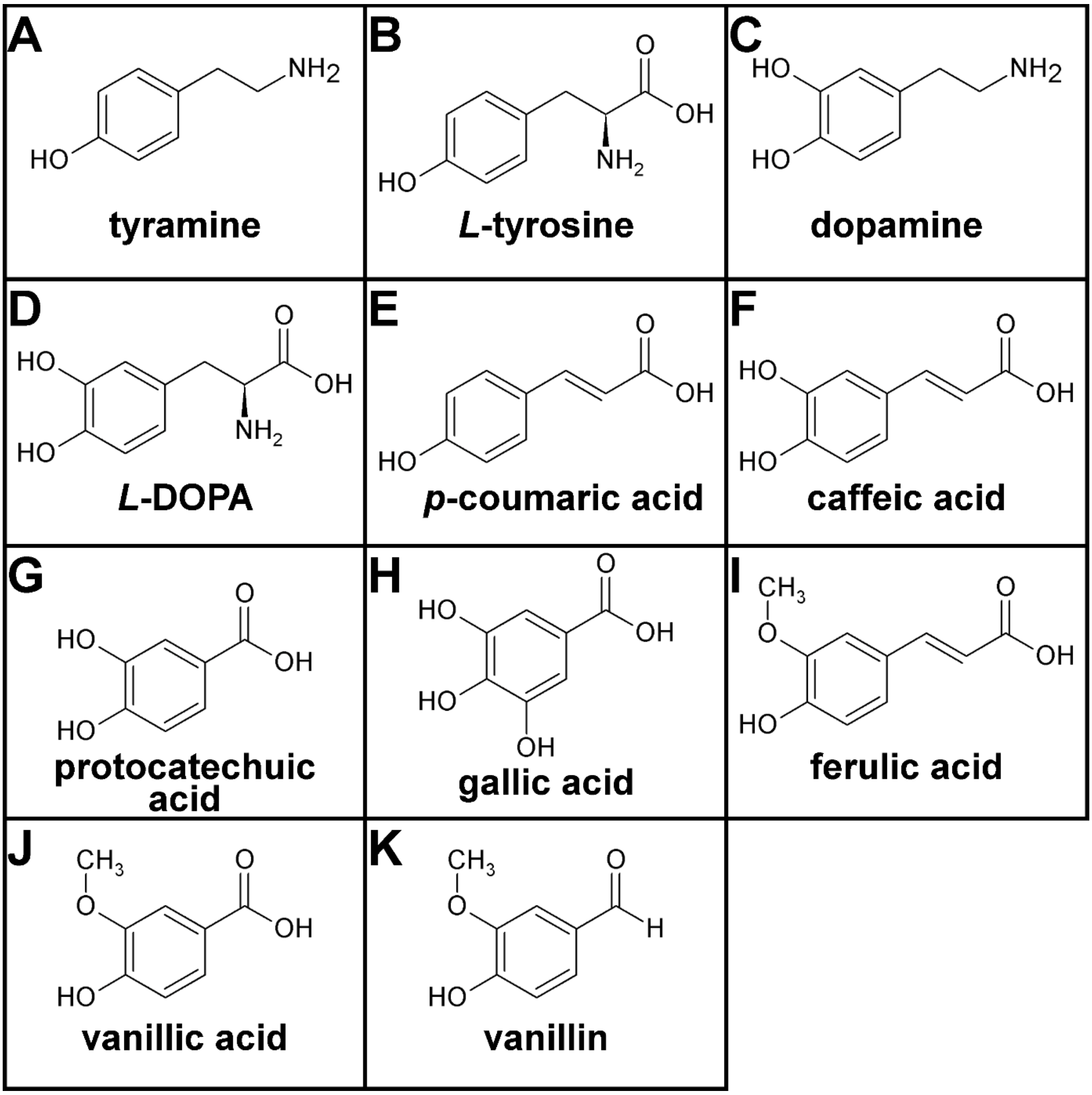

Figure S7. Structures of phenolic substrates. A = tyramine, **B** = *L*-tyrosine, **C** = dopamine, **D** = *L*-DOPA, **E** = *p*-coumaric acid, **F** = caffeic acid, **G** = protocatechuic acid, **H** = gallic acid, **I** = ferulic acid, **J** = vanillic acid, **K** = vanillin. The Figure has been edited using GIMP 2.10.18 (https://www.gimp.org).

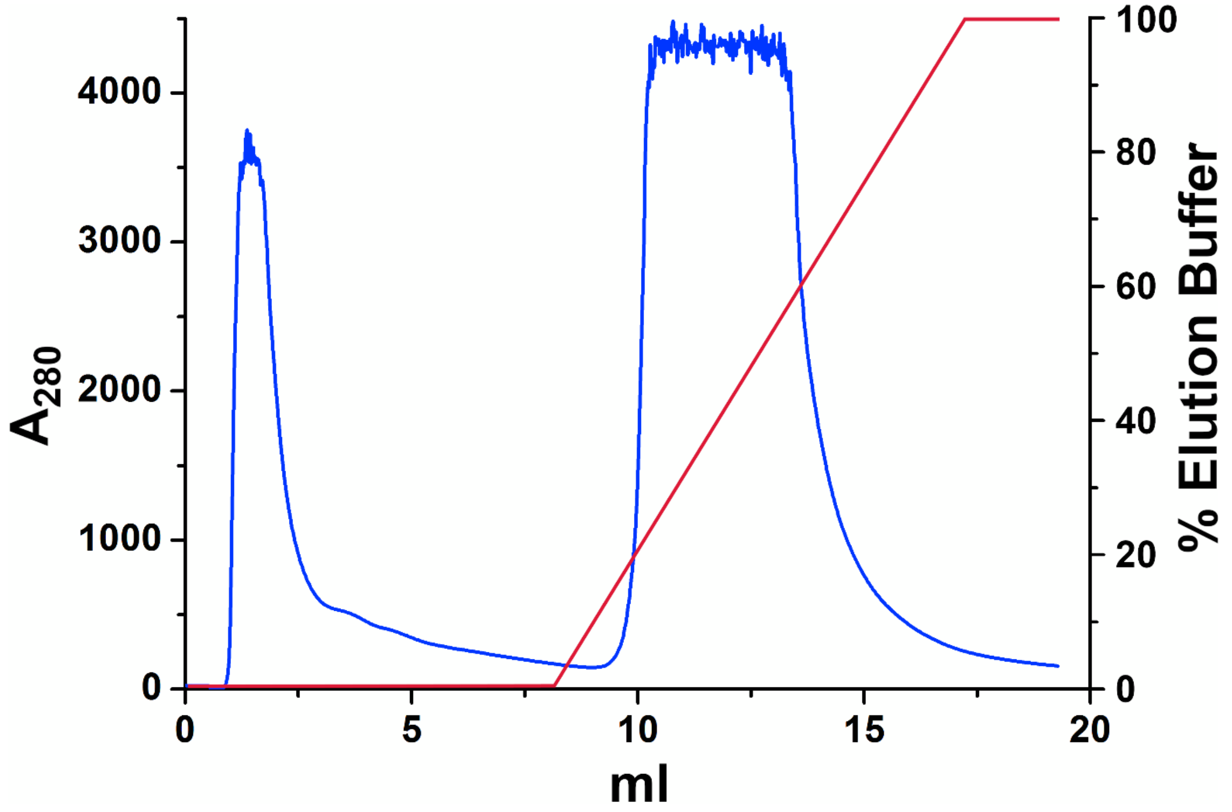

Figure S8. Chromatogram of the purification of *Sz*TYR via anion exchange chromatography. The blue line represents the absorption (mAU) at 280 nm. The red line represents the percentage of elution buffer. Start buffer: 10 mM Tris-HCl, pH 7.5; elution buffer: 2 M NaCl, 10 mM Tris-HCl, pH 7.5. After applying the sample to the column, the target protein (*Sz*TYR) passes through while contaminations bind to the column and elute only with increasing molarities of NaCl. A MonoQ column (1 ml column volume) has been used. Detailed information about the experimental setup is provided in the Materials and methods section. The Figure has been created using the OriginPro 8 software and was edited using GIMP 2.10.18 (https://www.gimp.org).

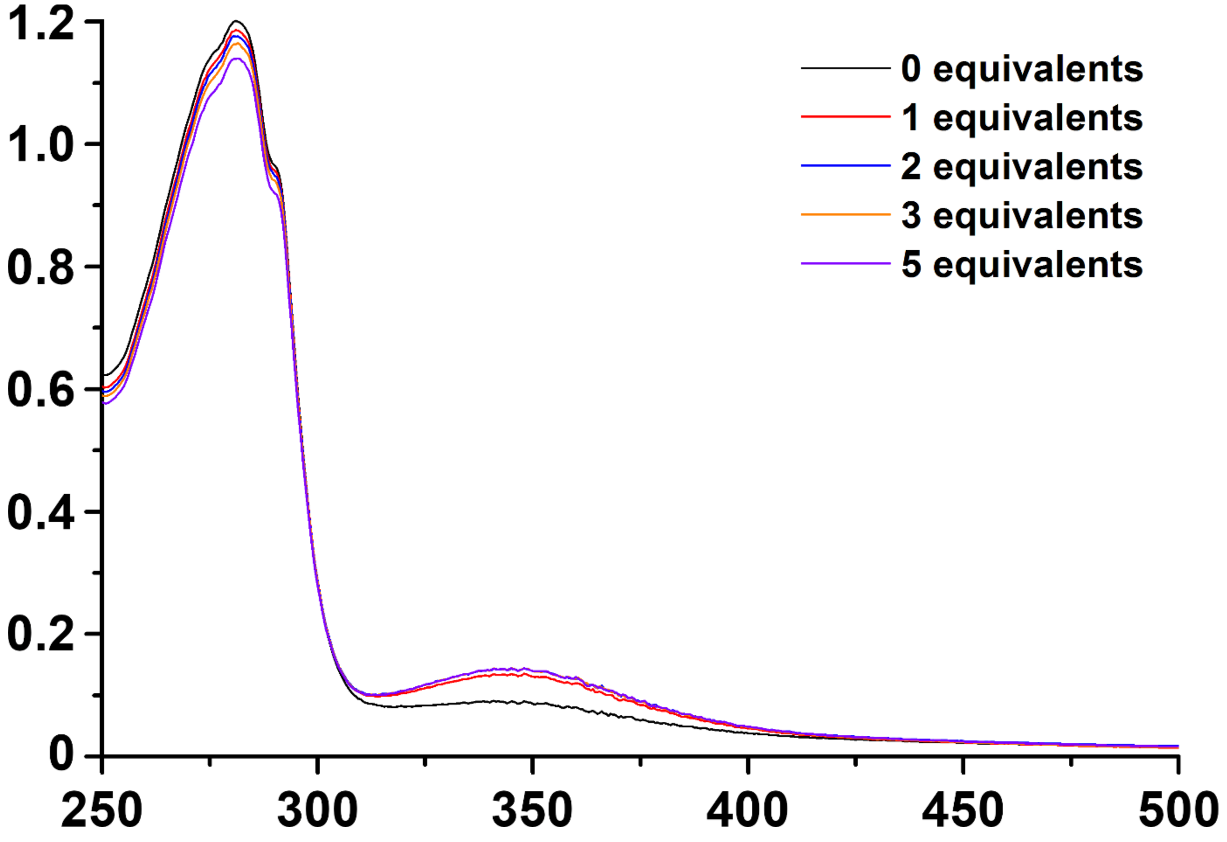

Figure S9. UV-Vis absorption spectrum of a 0.5 g/l solution of *Sz*TYR. A clear absorption shoulder at 295 nm is visible due to the presence of twelve tryptophan residues in the amino acid sequence. The absorption spectra were recorded after the addition of increasing molar equivalents of H_2O_2 (compared to *Sz*TYR). The addition of increasing amounts of H_2O_2 leads to the increased formation of an absorption band at ~ 345 nm, which corresponds to the formation of a charge-transfer transition band caused by dioxygen binding to the di-copper center. The charge-transfer band reaches saturation after the addition of 1 – 2 molar equivalents of H_2O_2 (ε = 8864 M^{-1} cm^{-1}). Spectra were recorded in 10 minutes intervals. The Figure has been created using the OriginPro 8 software edited using GIMP 2.10.18 (https://www.gimp.org).

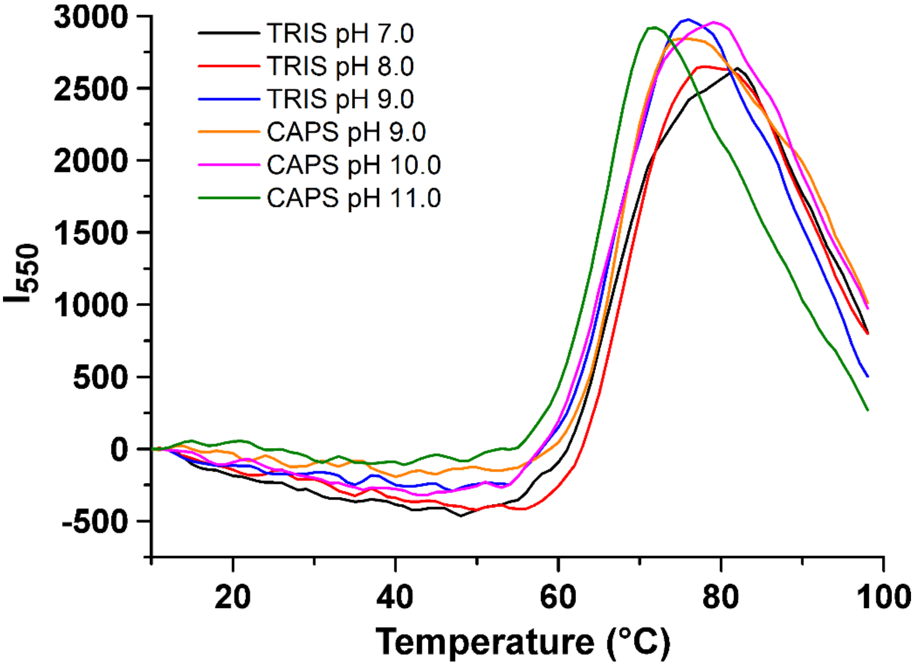

Figure S10. Thermofluor assay of *Sz*TYR at different pH values. The sigmoidal parts of the curves were used to determine T_m values (see supporting Materials and methods). Melting temperatures (T_m) are reported in Table S9. Measurements were performed in triplicates for each pH condition. For clarity, averaged fluorescence curves are presented.

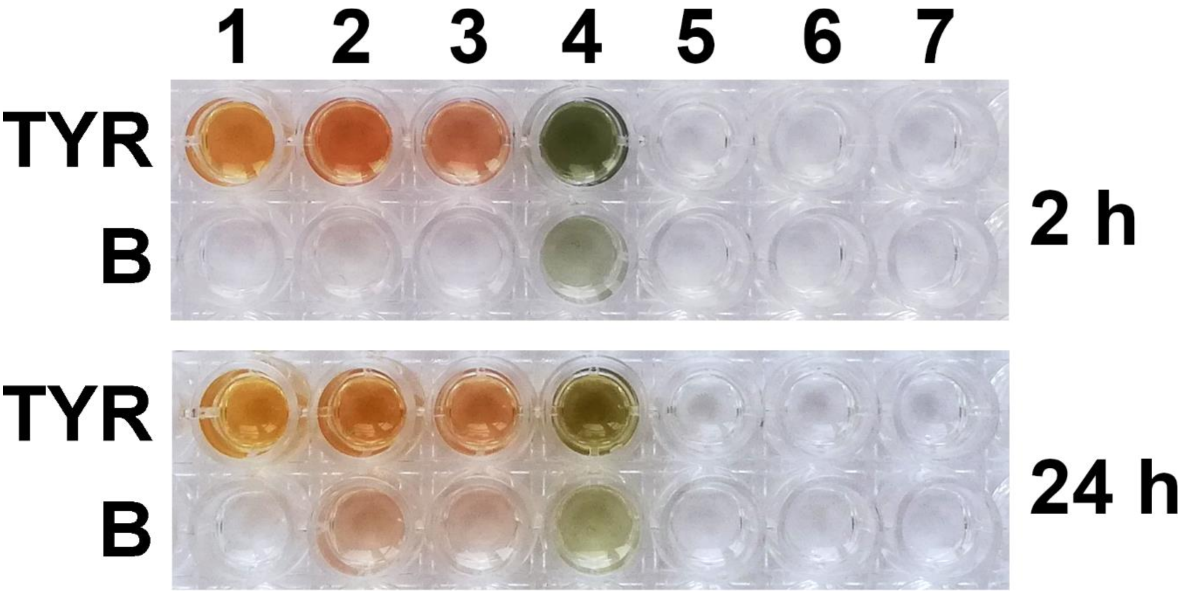

Figure S11. Substrate scope assay of *Sz*TYR using phenolic compounds naturally abundant in peatlands. **1** = coumaric acid, **2** = caffeic acid, **3** = protocatechuic acid, **4** = gallic acid, **5** = ferulic acid, **6** = vanillic acid, **7** = vanillin. *Sz*TYR has been added to lanes labeled TYR. Lanes labeled B represent the blank (no *Sz*TYR added). Detailed information about the

experimental setup is provided in the Materials and methods section of the main manuscript. The Figure has been edited using GIMP 2.10.18 (https://www.gimp.org).

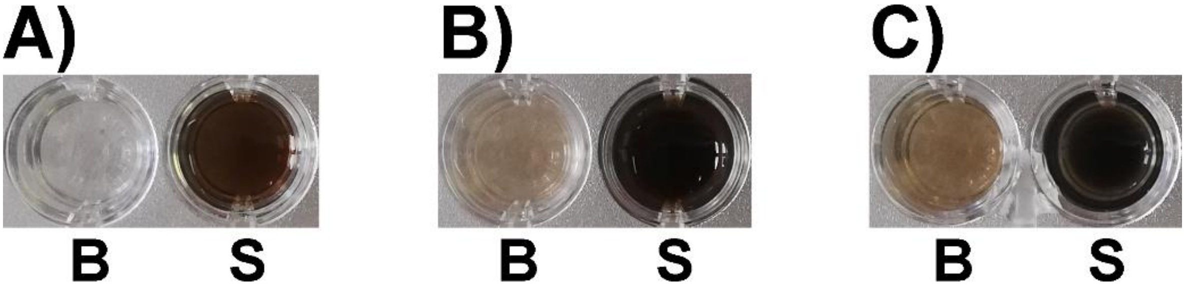

Figure S12. Co-polymerization of various phenolic substrates. A mixture of 2 mM coumaric acid, 2 mM caffeic acid, 2 mM protocatechuic acid and 2 mM gallic acid was incubated with 20 µg *Sz*TYR in a total volume of 200 µl and incubated for 72 hours. Photos were taken after **A)** 24 hours, **B)** 48 hours and **C)** 72 hours. S represents the sample (phenol-mix with *Sz*TYR), B represents the blank (phenol-mix without *Sz*TYR).

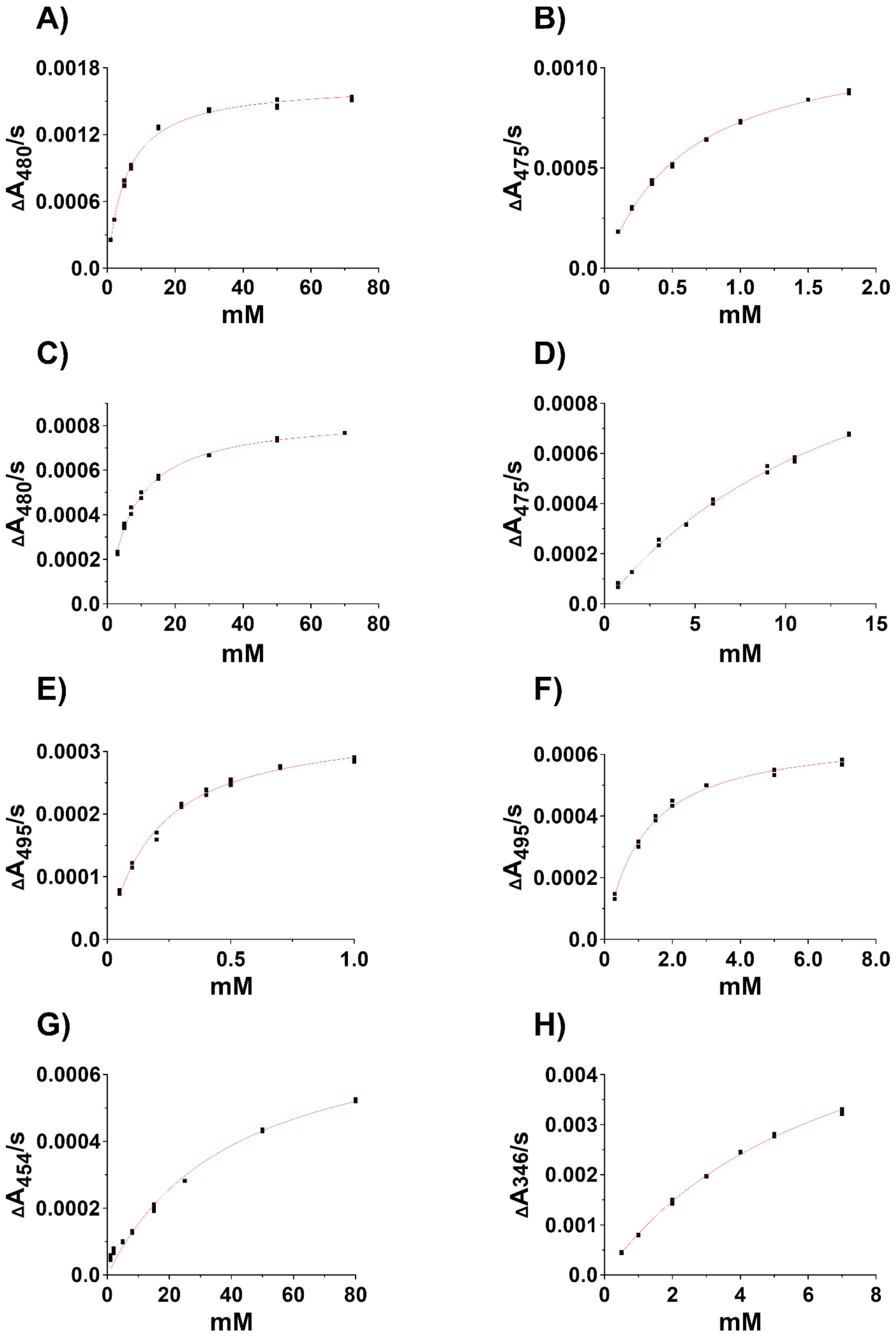

A)
0.0018
0.0012
0.0006
0.0
ΔA_{480}/s
0
20
40
60
80
mM
B)
0.0010
0.0005
0.0
ΔA_{475}/s
0
0.5
1.0
1.5
2.0
mM
C)
0.0008
0.0006
0.0004
0.0002
0.0
ΔA_{480}/s
0
20
40
60
80
mM
D)
0.0008
0.0006
0.0004
0.0002
0.0
ΔA_{475}/s
0
5
10
15
mM
E)
0.0003
0.0002
0.0001
0.0
ΔA_{495}/s
0
0.5
1.0
mM
F)
0.0006
0.0004
0.0002
0.0
ΔA_{495}/s
0
2.0
4.0
6.0
8.0
mM
G)
0.0006
0.0004
0.0002
0.0
ΔA_{454}/s
0
20
40
60
80
mM
H)
0.004
0.003
0.002
0.001
0.0
ΔA_{346}/s
0
2
4
6
8
mM

Figure S13. Non-liner curve fitting of data points measured for *Sz*TYR during kinetic assays. Triplets were fitted using the Hill-equation and the least-squares method implemented in the OriginPro 8 software. **A** = tyramine, **B** = *L*-tyrosine, **C** = dopamine, **D** = *L*-DOPA, **E** = *p*-coumaric acid, **F** = caffeic acid, **G** = protocatechuic acid, **H** = gallic acid. Detailed information about the experimental setup is provided in the Materials and methods section. The Figure has been created using the OriginPro 8 software and was edited using GIMP 2.10.18 (https://www.gimp.org).

5. References

(1) Porebski, S.; Bailey, G.; Baum, B. Modification of a CTAB DNA Extraction Protocol for Plants Containing High Polysaccharide and Polyphenol Components. *Plant Mol. Biol. Report.* **1997**, *15* (1), 8–15. DOI 10.1210/en.2002-220569.

(2) Stevenson, F. J. Humus Chemistry: Genesis, Composition, Reactions, 2nd Edn. *Wiley, New York.*; **1994**.

(3) Matheson, C. D.; Gurney, C.; Esau, N.; Lehto, R. Assessing PCR Inhibition from Humic Substances. *Open Enzym. Inhib. J.* **2010**, *3*, 38–45. DOI 10.2174/1874940201003010038.

(4) Roslan, M. A. M.; Mohamad, M. A. N.; Omar, S. M. High-Quality DNA from Peat Soil for Metagenomic Studies: A Minireview on DNA Extraction Methods. *Sci. Herit. J.* **2017**, *1*, 1–6. DOI 10.26480/gws.02.2017.01.06.

(5) Solano, F. Melanins: Skin Pigments and Much More—Types, Structural Models, Biological Functions, and Formation Routes. *New J. Sci.* **2014**, 498276. DOI 10.1155/2014/498276.

(6) Varadachari, C.; Ghosh, K. On Humus Formation. *Plant Soil* **1984**, *77*, 305–313. DOI 10.1007/BF02182933.

(7) Ito, S.; Sugumaran, M.; Wakamatsu, K. Chemical Reactivities of Ortho-Quinones Produced in Living Organisms: Fate of Quinonoid Products Formed by Tyrosinase and Phenoloxidase Action on Phenols and Catechols. *Int. J. Mol. Sci.* **2020**, *21*, 6080. DOI 10.3390/ijms21176080.

(8) Krachler, R.; von der Kammer, F.; Jirsa, F.; Süphandag, A.; Krachler, R. F.; Plessl, C.; Vogt, M.; Keppler, B. K.; Hofmann, T. Nanoscale Lignin Particles as Sources of Dissolved Iron to the Ocean. *Global Biogeochem. Cycles* **2012**, *26*, GB3024; DOI 10.1029/2012GB004294.

(9) Tarnawski, M.; Depta, K.; Grejciun, D.; Szelepin, B. HPLC Determination of Phenolic Acids and Antioxidant Activity in Concentrated Peat Extract - A Natural Immunomodulator. *J. Pharm. Biomed. Anal.* **2006**, *41*, 182–188; DOI 10.1016/j.jpba.2005.11.012.

(10) Ainsworth, E. A.; Gillespie, K. M. Estimation of Total Phenolic Content and Other Oxidation Substrates in Plant Tissues Using Folin-Ciocalteu Reagent. *Nat. Protoc.* **2007**, *2*, 875–877. DOI 10.1038/nprot.2007.102.

(11) Huynh, K.; Partch, C. L. Analysis of Protein Stability and Ligand Interactions by Thermal Shift Assay. *Curr. Protoc. Protein. Sci.* **2015**, *79*, 28.9.1–28.9.14. DOI 10.1002/0471140864.ps2809s79.

(12) Sievers, F.; Wilm, A.; Dineen, D.; Gibson, T. J.; Karplus, K.; Li, W.; Lopez, R.; McWilliam, H.; Remmert, M.; Söding, J.; Thompson, J. D.; Higgins, D. G. Fast, Scalable Generation of High-Quality Protein Multiple Sequence Alignments Using Clustal Omega. *Molecular systems biology*. **2011**, *7*, 539. DOI 10.1038/msb.2011.75.

(13) Pearson, W. R. Selecting the Right Similarity-Scoring Matrix. *Curr Protoc Bioinforma.* **2013**, *43*, 3.5.1–3.5.9. DOI 10.1002/0471250953.bi0305s43.

(14) Kumar, S.; Stecher, G.; Li, M.; Knyaz, C.; Tamura, K. MEGA X: Molecular Evolutionary Genetics Analysis across Computing Platforms. *Mol. Biol. Evol.* **2018**, *35*, 1547–1549. DOI 10.1093/molbev/msy096.

(15) Umek, N.; Geršak, B.; Vintar, N.; Šoštarič, M.; Mavri, J. Dopamine Autoxidation Is Controlled by Acidic PH. *Front. Mol. Neurosci.* **2018**, *11*, 467. DOI 10.3389/fnmol.2018.00467.

(16) Muñoz, J. L.; García-Molina, F.; Varón, R.; Rodriguez-Lopez, J. N.; García-Cánovas, F.; Tudela, J. Calculating Molar Absorptivities for Quinones: Application to the Measurement of Tyrosinase Activity. *Anal. Biochem.* **2006**, *351*, 128–138. DOI 10.1016/j.ab.2006.01.011.

(17) Panis, F.; Rompel, A. Identification of the Amino Acid Position Controlling the Different Enzymatic Activities in Walnut Tyrosinase Isoenzymes (*Jr*PPO1 and *Jr*PPO2). *Sci. Rep.* **2020**, *10*, 10813. DOI 10.1038/s41598-020-67415-6.

(18) Sendovski, M.; Kanteev, M.; Ben-Yosef, V. S.; Adir, N.; Fishman, A. First Structures of an Active Bacterial Tyrosinase Reveal Copper Plasticity. *J. Mol. Biol.* **2011**, *405*, 227–237. DOI 10.1016/j.jmb.2010.10.048.
